# GRaNIE and GRaNPA: inference and evaluation of enhancer‐mediated gene regulatory networks

**DOI:** 10.15252/msb.202311627

**Published:** 2023-04-19

**Authors:** Aryan Kamal, Christian Arnold, Annique Claringbould, Rim Moussa, Nila H Servaas, Maksim Kholmatov, Neha Daga, Daria Nogina, Sophia Mueller‐Dott, Armando Reyes‐Palomares, Giovanni Palla, Olga Sigalova, Daria Bunina, Caroline Pabst, Judith B Zaugg

**Affiliations:** ^1^ European Molecular Biology Laboratory, Structural and Computational Biology Unit Heidelberg Germany; ^2^ Faculty of Biosciences Collaboration for Joint PhD Degree between EMBL and Heidelberg University Heidelberg Germany; ^3^ Department of Medicine V, Hematology, Oncology and Rheumatology University Hospital Heidelberg Heidelberg Germany; ^4^ Molecular Medicine Partnership Unit University of Heidelberg Heidelberg Germany; ^5^ Present address: Department of Biochemistry and Molecular Biology Complutense University of Madrid Madrid Spain; ^6^ Present address: Institute of Computational Biology Helmholtz Center Munich Oberschleißheim Germany

**Keywords:** enhancers, gene regulatory networks, macrophage biology, multiomics data integration, transcriptional regulation, Chromatin, Transcription & Genomics, Computational Biology

## Abstract

Enhancers play a vital role in gene regulation and are critical in mediating the impact of noncoding genetic variants associated with complex traits. Enhancer activity is a cell‐type‐specific process regulated by transcription factors (TFs), epigenetic mechanisms and genetic variants. Despite the strong mechanistic link between TFs and enhancers, we currently lack a framework for jointly analysing them in cell‐type‐specific gene regulatory networks (GRN). Equally important, we lack an unbiased way of assessing the biological significance of inferred GRNs since no complete ground truth exists. To address these gaps, we present GRaNIE (Gene Regulatory Network Inference including Enhancers) and GRaNPA (Gene Regulatory Network Performance Analysis). GRaNIE (https://git.embl.de/grp‐zaugg/GRaNIE) builds enhancer‐mediated GRNs based on covariation of chromatin accessibility and RNA‐seq across samples (e.g. individuals), while GRaNPA (https://git.embl.de/grp‐zaugg/GRaNPA) assesses the performance of GRNs for predicting cell‐type‐specific differential expression. We demonstrate their power by investigating gene regulatory mechanisms underlying the response of macrophages to infection, cancer and common genetic traits including autoimmune diseases. Finally, our methods identify the TF PURA as a putative regulator of pro‐inflammatory macrophage polarisation.

## Graphical abstract

GRaNIE builds enhancer‐based gene regulatory networks (GRNs) using chromatin accessibility and RNA‐seq data. GRaNPA assesses the biological significance of GRNs and transcription factors. Together, they provide insights into cell‐type‐specific gene regulation.

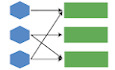

## Introduction

Enhancers are genomic locations that play an important role in cell‐type‐specific gene regulation, and impaired enhancer function has been linked to an increasing number of diseases (Karnuta & Scacheri, 2018; Claringbould & Zaugg, 2021). In particular, genome‐wide association studies (GWAS) have linked over 200,000 common genetic variants with over 40,000 traits and diseases. Since the vast majority of these disease‐associated genetic variants lie in noncoding regions far from promoters (Claringbould & Zaugg, 2021), they are likely affecting enhancers and having a regulatory role.

A big challenge in the post‐GWAS era is the interpretation of these disease‐associated genetic variants in noncoding genomic regions because it is often still unclear what genes they target, and in what cell types. The cell‐type‐specific activity of gene regulatory elements is likely conferred by transcription factors (TFs). And indeed, a recent study points at the importance of studying TFs for understanding genetic variants associated with autoimmune diseases (Freimer *et al*, 2022). The importance of TFs was confirmed by another study, which found that *trans‐*expression quantitative trait loci (eQTLs), which likely act via TFs, are more enriched in disease‐associated genes than *cis*‐eQTLs (Võsa *et al*, 2021). However, to predict the function of TFs, for example in cell‐fate determination, it is crucial to include putative enhancers (preprint: Janssens *et al*, 2021; Xu *et al*, 2021). Enhancer‐mediated gene regulatory networks reconstructed from single‐cell RNA and ATAC‐seq profiling in the fly brain have led to a better understanding of the regulatory diversity across different neuronal cell types (preprint: Janssens *et al*, 2021). We previously used enhancer‐based analyses to understand disease mechanisms in pulmonary arterial hypertension (Reyes‐Palomares *et al*, 2020). Thus, for interpreting disease‐associated genetic variants, or enhancers in general, it is crucial to jointly investigate TF activity, enhancers and gene expression in a cell‐type‐specific manner.

Several approaches have been proposed to infer bipartite TF‐gene networks, for example based on co‐expression in bulk (Huynh‐Thu *et al*, 2010; Haynes *et al*, 2013), or single‐cell expression data (Aibar *et al*, 2017; Moerman *et al*, 2019; preprint: Kamimoto *et al*, 2020), based on partial information decomposition (Chan *et al*, 2017), time‐course data (Huynh‐Thu & Geurts, 2018) or data curation (Liu *et al*, 2015; Han *et al*, 2018; Garcia‐Alonso *et al*, 2019; Keenan *et al*, 2019). At the same time, methods for inferring enhancer‐gene links exist, for example using co‐variation of peaks (Pliner *et al*, 2018; Fulco *et al*, 2019), or targeted perturbations of enhancers followed by sequencing (Schraivogel *et al*, 2020). Only few approaches jointly infer TF‐enhancer and enhancer‐gene links (Marbach *et al*, 2016) mostly from single‐cell data (preprint: González‐Blas *et al*, 2022; Fleck *et al*, 2022) and we currently lack methods to infer cell‐type‐specific networks that enable the study of context‐specific interaction between TFs, regulatory elements and genes.

An important step in regulatory network reconstruction is to evaluate their biological significance. Common approaches for assessing regulatory interactions include benchmarking against networks from simulated data or against known biological networks (Chen & Mar, 2018; Pratapa *et al*, 2020). Each of these has their own drawback: simulated networks are based on many assumptions about the network structure which may not reflect the “true” biological network while known biological networks typically suffer from a strong literature bias (Weidemüller *et al*, 2021), low complexity and a limited range of connections and cell types, and are thus not well‐suited for an unbiased evaluation of GRNs. In general, each network inference method will have its own bias and shortcomings, and performance will depend on the benchmarking data set (Chen & Mar, 2018; Pratapa *et al*, 2020). Thus, there is a need for an unbiased approach to assess the biological relevance of inferred and curated regulatory interactions as well as individual TF regulons (defined as all genes connected to a TF).

Here, we present a tool‐suite for building and evaluating enhancer‐based gene regulatory networks (eGRNs) called GRaNIE (Gene Regulatory Network Inference including Enhancers ‐ https://grp‐zaugg.embl‐community.io/GRaNIE and https://bioconductor.org/packages/GRaNIE) and GRaNPA (Gene Regulatory Network Performance Analysis ‐ https://git.embl.de/grp‐zaugg/GRaNPA), respectively. GRaNIE jointly infers TF‐enhancer and enhancer‐gene interactions based on covariation of bulk RNA‐seq expression and chromatin accessibility (ATAC‐seq) or ChIP‐seq for active histone marks (e.g. H3K27ac) across biological samples. GRaNPA assesses the biological relevance—of any TF‐gene‐based GRN—using a machine learning framework, and identifies TFs that predict cell‐type‐specific expression response to perturbations. We demonstrate that GRaNIE infers biologically meaningful eGRNs using macrophages as example, and validate TF‐enhancer links with ChIP‐seq and enhancer‐gene links with eQTL data. We further demonstrate the cell‐type‐specific nature of GRaNIE‐inferred eGRNs for macrophages, T‐cells and acute myeloid leukaemia (AML) cells using GRaNPA evaluation, and by predicting cell‐type‐specific TF knockout (K/O) data. Using GRaNIE followed by GRaNPA, we identify PURA as putative TF driving the pro‐inflammatory polarisation of macrophages, which we corroborate with orthogonal phosphoproteomics data, and we confirm earlier observations from mice that MBD2 drives the anti‐inflammatory program in macrophages. Furthermore, we find enhancers in the macrophage eGRNs enriched for autoimmune disease variants, which GRaNIE links to upstream TFs and putative target genes. Finally, we provide a comprehensive resource of cell‐type‐specific GRNs for three other cell types (https://apps.embl.de/grn/).

## Results

### Overview and conceptual description of the GRaNIE algorithm

We developed GRaNIE to interpret genetic and epigenetic variation in regulatory (enhancer and promoter) regions, here defined by ATAC‐seq peaks and hereafter referred to as “peaks”. GRaNIE is an R/Bioconductor package and jointly infers TF‐enhancer/promoter and enhancer/promoter‐gene interactions from the same data in a context‐specific manner. Conceptually, the software is based on an approach we have devised for a recent study in which we investigated enhancer‐mediated disease mechanisms of pulmonary arterial hypertension (Reyes‐Palomares *et al*, 2020). Briefly, GRaNIE separately identifies TF‐peak and peak‐gene links, and then integrates them into an eGRN.

The TF‐peak links are based on statistically significant co‐variation of TF expression and peak accessibility across samples (e.g. individuals, recommended minimum number ~10–15), taking into account predicted TF binding sites. To obtain them, GRaNIE calculates all pairwise correlations between TF expression levels (RNA‐seq) and peak signal (ATAC/ChIP‐seq), stratified by whether the peak overlaps a predicted binding site of the TF. For each TF, it then uses the distribution of all peaks that do not contain its predicted binding site as background to calculate an empirical FDR for assessing the significance of TF‐peak links (Appendix Fig S1). In our previous work, we have demonstrated that negative TF‐peak correlations indicate the TF acts as transcriptional repressor while positive correlations indicate an activator role, thus allowing the classification of TFs into activators and repressors (Berest *et al*, 2019). As a quality control (QC), we recommend comparing the number of real TF‐peak links to those obtained from a background set of links inferred from randomised data (see Materials and Methods).

**Figure 1:**
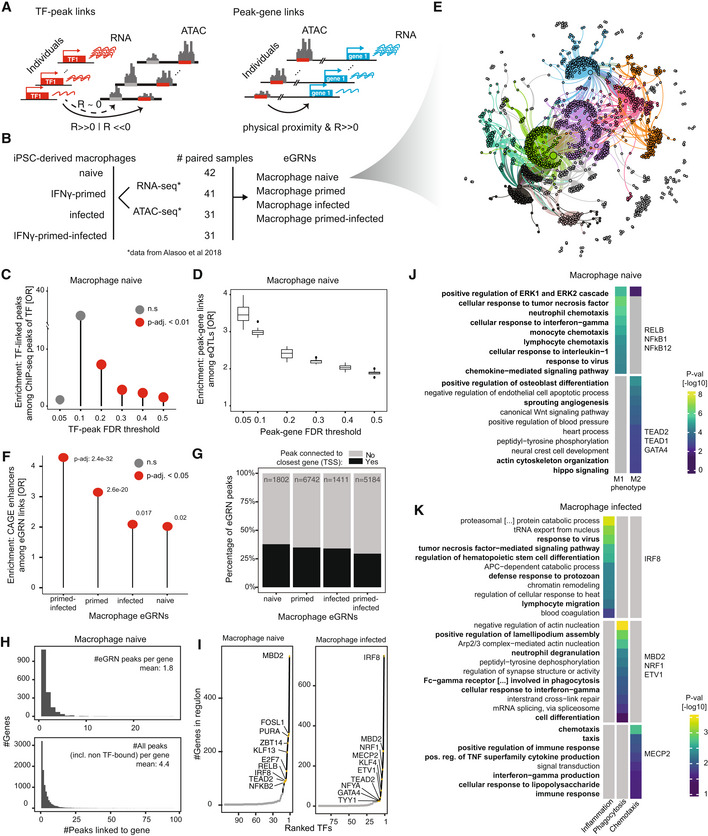
Overview, application and validation of GRaNIE ASchematic of the eGRN construction by GRaNIE, including the TF to peak (left) and peak to gene (right) links (detailed workflow in Appendix Fig S1).BDatasets used for macrophage eGRN construction and evaluation.CValidation of the eGRN TF‐peak links with ChIP‐seq data. Enrichment of ChIP‐seq peaks overlapping a GRaNIE‐inferred TF‐bound peak (same TF) are shown for different TF‐peak FDRs in the naive macrophage eGRN. Statistical significance was determined using Fishers Exact test; test set: all TF‐peak pairs where the peak contains the motif for the respective TF (*n* = 25,205, 39,408, 78,971, 109,228, 142,548, 147,226 for TF‐peak FDR 0.05, 0.1, 0.2, 0.3, 0.4, 0.5 respectively), categories: overlap with ChIP‐seq signal, part of GRaNIE‐infer network. Only TFs for which ChIP‐seq data was available are considered (see Appendix Fig S2 for other eGRNs).DValidation of the eGRN peak‐gene links with macrophage eQTLs. Plots show the enrichment of eGRN links overlapping an eQTL over randomly sampled distance‐matched peak‐gene links for different peak‐gene FDRs in the naive macrophage eGRN (see Appendix Fig S3 for other eGRNs). Boxplots: central band: 50% quantile, box: interquartile range (25–75%); whiskers: max/min are 1.5 IQR above/below the box.EForce‐directed visualisation of the naive macrophage eGRN (see Appendix Fig S4 for the other eGRNs). The colours correspond to the identified communities.FEnrichment of macrophage‐specific FANTOM5 CAGE enhancers among the macrophage eGRN peaks. Statistical significance was determined with Fisher's exact test; test set: all peaks that were considered for peak‐gene connections (ATAC consensus peaks located within 250 kb of a TSS of a gene with mean normalised expression across samples > 1) in each eGRN (*n* = 210,083, 227,035, 227,120 and 219,823 peaks for the naive, infected, primed and primed‐infected eGRN, respectively), categories: overlap with CAGE enhancer, part of GRaNIE network.GFraction of eGRN peaks connected to the closest gene (black) versus other (grey) genes for the macrophage eGRNs.HNumber of peaks linked to a gene shown as histogram for eGRN peaks (top) and all peaks (including non‐TF bound; bottom) for the naive macrophage data (see Appendix Fig S7 for other eGRNs). Mean number of peaks indicated in the panels.INumber of genes connected to each TF for the naive macrophage eGRN (top 10 TFs are labelled).J, KGO enrichment and associated *P*‐values for selected communities from the naive (J) and infected (K) macrophage eGRN (see Dataset EV7 for the full table of enrichments across communities for all macrophage eGRNs).

Peak‐gene links are based on significant co‐variation of peak accessibility and gene expression across samples (Fig 1A, Appendix Fig S1). For this, GRaNIE calculates the correlation between the expression of a gene and signals of all peaks within an adjustable, defined distance of its transcription start site (TSS, default is 250 kb). GRaNIE also allows the use of chromatin conformation data, such as Hi‐C, to define which peak‐gene pairs will be tested within a 3D proximity (Appendix Fig S1). Since chromatin accessibility in regulatory elements is generally associated with active gene regulation and transcription, we only expect positive correlations for functional peak‐gene links. Notably, this is still true for repressor‐bound elements, where binding of most repressors leads to loss of both accessibility and transcription (Berest *et al*, 2019). Negative peak‐gene correlations have no clear biological meaning and may indicate remaining batch effects or random noise. Therefore, one can judge the signal‐to‐noise ratio by assessing positive versus negative peak‐gene correlations and we implemented this as a QC metric in GRaNIE. We recommend comparing these QC metrics with a corresponding background set (implemented in GRaNIE, see Materials and Methods).

GRaNIE then combines TF‐peak and peak‐gene links to create a tripartite TF‐enhancer‐gene eGRN, based on three user‐defined thresholds: FDR of TF‐enhancer edges, FDR of enhancer‐gene edges and maximum distance between enhancer and TSS.

The whole framework is implemented in a user‐friendly R/Bioconductor package. In addition to the eGRN reconstruction function, GRaNIE comprises a set of easy‐to‐use functions for generating and visualising network statistics, identifying network communities, performing subnetwork‐specific Gene Ontology (GO) enrichment analysis and various QC plots. An extensive documentation is available at (https://grp‐zaugg.embl‐community.io/GRaNIE).

### Application of GRaNIE for generating cell‐type‐specific eGRN in macrophages

Macrophages are large white blood cells of the innate immune system that can be found in essentially all tissues. They play a role in inflammatory disorders and genetic variants associated with several autoimmune and other diseases are enriched for enhancers active in macrophages (Alasoo *et al*, 2018; Novikova *et al*, 2021). Given that inflammatory conditions are underlying many common diseases, macrophages are an important cell type within which disease‐associated variants manifest their effect. Thus, macrophages present an ideal cell type to apply and test the eGRN framework.

We obtained paired RNA‐ and ATAC‐seq data for induced pluripotent stem cell (iPSC) derived macrophages from 31 to 45 individuals in four conditions (naive, interferon gamma (IFN‐γ)‐primed, infected (with Salmonella), and IFN‐γ‐primed‐infected) (Alasoo *et al*, 2018) (Fig 1B). For each of these, we run GRaNIE using TF‐binding sites predictions based on HOCOMOCO v11 and PWMScan as described previously (Berest *et al*, 2019).

We next assessed the GRaNIE‐inferred eGRNs using independent molecular evidence, and used this to define reasonable default values for the TF‐peak and peak‐gene FDR thresholds. Since molecular ground truth data for TF‐peak‐gene links does not exist, we evaluated each type of link independently using cell‐type‐specific ChIP‐seq data for the TF‐peak links and cell‐type‐specific eQTL data for the peak‐gene links. Specifically, we obtained macrophage‐specific ChIP‐seq data from ReMap 2022 (Hammal *et al*, 2022), and quantified the enrichment of GRaNIE‐inferred TF‐bound peaks among ChIP‐seq peaks using ATAC‐peaks that contain the TF motif as background (see Materials and Methods). For the naive, the primed and the infected macrophage eGRNs, this revealed a significant enrichment for ChIP‐seq signal at FDR 0.2 that steadily decreased with increasing FDR (Fig 1C, Appendix Fig S2). The primed‐infected eGRN did not show any significant enrichment, so we excluded this eGRN from further analyses. For the peak‐gene links, we used macrophage‐specific *cis*‐eQTLs to assess the enrichment of eQTL links in the GRaNIE links over a distance‐matched set of control links at various FDRs. This revealed a steady decrease in the odds ratio with increasing FDR for all three remaining eGRNs (Fig 1D, Appendix Fig S3). Based on these results, we chose 0.2 as default for TF‐peak FDR and 0.1 as default for peak‐gene FDR.

Using these default parameters, we obtained an eGRN for each of the three conditions comprising 92–126 TFs, 1,411–6,742 enhancers, and 1,454–3,869 genes (Fig 1E, Appendix Fig S4A–C; Table 1 and Datasets [Link], [Link]). For all eGRNs, we observed much fewer significant connections when running GRaNIE on randomised data (permuted sample labels, peak labels and motif labels; Appendix Fig S5A–D), signifying that their TF‐peak links pass QC. Similarly, for the peak‐gene links, we find that all eGRNs show more signal for positive (expected signal) than negative (noise) correlations, and that the signal‐to‐noise ratio decreases with peak‐gene distance until no signal is left for random peak‐gene pairs (Appendix Fig S6A–D). In addition, we observed a significant enrichment for the TF‐peak‐gene links among cell‐type‐specific active enhancers based on CAGE data (Andersson *et al*, 2014) (Fig 1F), further corroborating that GRaNIE infers biologically meaningful eGRNs.

**Table 1:** Summary of the eGRNs described in the main text.

eGRN	# TFs (activators, repressors)	# peaks	# genes	# connections TF‐enhancer‐genes
Macrophage naive (naive)	114 (52, 28)	1,802	1,793	3,209
Macrophage IFN‐γ primed (primed)	126 (65, 31)	6,742	3,869	22,082
Macrophage infected with Salmonella (infected)	92 (35, 26)	1,411	1,454	2,128
Macrophage IFN‐γ‐primed and infected (primed‐infected)	78 (30, 22)	5,184	2,732	14,697
AML	53 (30, 4)	2,896	2,525	5,466
Primary CD4^+^ T‐cells	94 (20, 16)	3,469	3,258	8,920

Network statistics for the various eGRNs based on default parameters (TF‐peak FDR = 0.2, peak‐gene FDR = 0.1, peak‐gene distance ≤ 250 kb, activator/repressor stringency threshold based on the 10th percentile).

We observed a slightly larger number of TFs classified as activators than as repressors (1.5–2‐fold), yet activators were connected with more peaks resulting in over 10‐fold more peaks being linked to an activator than to a repressor (Appendix Fig S5A–D; Table 1). Notably, in all eGRNs, only about 20–30% of the peaks are linked to their closest gene TSS (Fig 1G), an observation that is consistent with previous observations in pulmonary arterial endothelial cells (Reyes‐Palomares *et al*, 2020) and iPSC‐derived cardiomyocytes (preprint: Bunina *et al*, 2021).

On average, a gene is linked to 4.4 (naive), 2.9 (infected) and 5.9 (primed) peaks, of which 1.8 (naive), 1.5 (infected) and 5.7 (primed) are TF‐bound and thus part of a GRaNIE eGRN (Fig 1H, Appendix Fig S7). This discrepancy suggests that we are still missing some TF‐peak interactions (see Box 1). The majority of TFs are connected to very few genes, yet a handful of TFs are connected to over 50 genes, as exemplified for the eGRN from naive and the infected macrophages (Fig 1I), which is in line with the typically scale‐free structure of GRNs (Ouma *et al*, 2018). The most connected TFs in the infected and the naive eGRNs include many well‐established macrophage TFs such as IRF8, NFKB2 and RELB (Grigoriadis *et al*, 1996; Langlais *et al*, 2016), as well as noncanonical macrophage TFs MBD2, FOSL1 and NRF1. The latter have only recently been implicated in macrophage biology in mouse studies (Morishita *et al*, 2009; An *et al*, 2020; Jones *et al*, 2020).

Box 1 **Limitations of GRaNIE**

- As with all network inference tools, it is important to keep in mind what an edge means. In the case of GRaNIE, the TF‐peak and peak‐gene links are based on co‐variation across biological samples (in this study variation across individuals). Therefore, it will miss links when either of the nodes (TF expression, peak accessibility, or gene expression) is not variable across samples. For instance, if samples are individuals, GRaNIE may miss house‐keeping and dosage‐sensitive genes, TFs, and enhancers if they are equally active between individuals.
- If GRaNIE is run with ATAC‐seq data, the limitations of ATAC‐seq apply: i.e. accessibility may not always reflect activity. Specifically, promoter accessibility is not necessarily correlated with gene expression. Therefore, GRaNIE will likely miss some promoter‐gene connections. Furthermore, it will not detect TFs that do not affect accessibility.
- As with most TF‐inference based tools, GRaNIE relies on the availability of a TF binding site within a peak. Therefore, it will miss TFs for which binding sites are unknown, and TFs binding events that do not rely on the TF motif (e.g. cooperative binding).
- TF expression is not always predictive of a TF's role in transcriptional regulation. To circumvent this, GRaNIE offers the option of using TF motif accessibility as an estimate of TF activity. This in turn has the caveat that connections will be based on TF motifs, which can be very similar across TFs.
- Since GRaNIE is association‐based, it cannot *per se* distinguish direct from indirect effects. This is particularly important when running it on samples that are very different (e.g. different cell types). It may then become difficult to assess whether the variation in peak accessibility is driven by the TF for which it has a motif, or by some other mechanism. We refer the users to the QC implemented in GRaNIE to judge the extent of such an existing batch effect.

To dive into the biological processes captured by eGRNs, GRaNIE provides functionalities for identifying subnetworks, or communities (using Louvain clustering by default, as implemented in the *igraph* package in R; Blondel *et al*, 2008), and performing GO term enrichment on them. In line with a scale‐free architecture of the networks, we typically observe a few large communities and a long tail of very small and isolated nodes for each eGRN (Appendix Fig S8). Among the communities (Appendix Fig S8A) of the naive macrophage eGRN, one is enriched for GO terms related to pro‐inflammatory processes (response to IL‐1, chemotaxis, response to IFN‐γ) and one for anti‐inflammatory processes (angiogenesis, cytoskeleton reorganisation, positive regulation of osteoblast differentiation; Fig 1J), recapitulating the potential of naive macrophages to polarise into either M1 (pro‐inflammatory) or M2 (anti‐inflammatory) cell states (Murray, 2017). We find the M1‐phenotype cluster regulated by NFKB1/2 and REL, while the M2‐phenotype cluster is regulated by TEAD1/2 and GATA4. Among the communities of the infected macrophage eGRN (Appendix Fig S8D), one was enriched for pro‐inflammatory processes, one for phagocytosis‐related processes, and one for chemotaxis (Fig 1K), thus recapitulating the most important facets of macrophage function (Nathan *et al*, 1983; Parameswaran & Patial, 2010; Meng *et al*, 2014). Notably, each of these functional communities was regulated by a specific set of TFs: IRF8 for the pro‐inflammatory community, MBD2, NFR1 and ETV1 for the phagocytosis, and MECP2 for the chemotaxis.

As utility evaluation of the GRaNIE eGRNs, we compared real versus permuted eGRNs in terms of number and biological specificity of GO terms enriched in the TF regulons. Notably, the regulons of the permuted networks had the same degree distribution and thus the size distribution of the regulons (see Materials and Methods). The regulons from the permuted networks were enriched in less specific GO terms unrelated to macrophage biology compared with the regulons of the real eGRN (Appendix Fig S9).

In summary, these results demonstrate that GRaNIE‐inferred eGRNs capture molecular evidence from eQTLs, ChIP‐seq and CAGE data, and are useful for investigating TF‐driven biological processes in a cell type/state‐specific manner. Limitations of GRaNIE are outlined in Box 1.

### Conceptual description of GRaNPA, an approach for evaluating the biological relevance of GRNs and TFs

The premise of our proposed GRN evaluation framework is that cell‐type‐specific GRNs should capture cell‐type‐specific changes in gene expression patterns that are driven by TFs. For this, we devised a machine learning approach, GRaNPA (Gene Regulatory Network Performance Analysis), which evaluates how well the bipartite TF‐gene connections of an eGRN can predict cell‐type‐specific differential gene expression. At the same time, this framework identifies the TFs that are important for the prediction.

**Figure 2:**
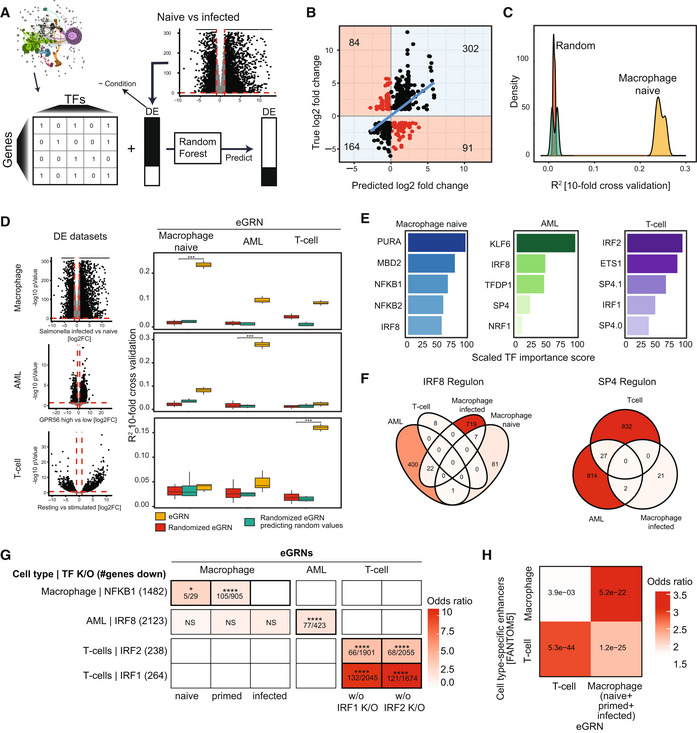
Overview and application of GRaNPA Schematic of the general GRN evaluation approach GRaNPA.Output of GRaNPA is shown as true versus predicted log2 fold‐changes for the macrophage expression response to Salmonella infection. Predictions are based on the naive macrophage eGRN (see Appendix Fig S10 for the other macrophage eGRNs).Output of GRaNPA is shown as density distribution of *R* ^2^ for 10 random forest runs for the naive macrophage eGRN predicting differential expression upon Salmonella infection, along with the two permuted controls.GRaNPA evaluation of eGRNs for naive macrophages (left), AML (middle) and T‐Cells (right) of differential expression from macrophages infected with Salmonella versus naive (top), two subtypes of AML (middle), and resting versus stimulated T‐cells (bottom). Red lines indicate the log2 fold‐change (vertical line) and *P*‐value (horizontal line) thresholds for genes included in the GRaNPA analysis. Distributions of *R* ^2^ from distinct random forest runs (*n* = 10) are shown as boxplots; *t*‐tests were performed to compare GRaNPA performance between the permuted and real networks (***P < 0.001). Boxplots: central band: 50% quantile, box: interquartile range (25–75%); whiskers: max/min are 1.5 IQR above/below the box.Top 5 most important TFs (0.0 and 0.1 indicate distinct TF motifs as defined by the HOCOMOCO database) for each of the eGRNs in (D) based on prediction in the same cell‐type.Overlap of SP4 (left) and IRF8 (right) regulons between eGRNs from different cell types (only eGRNs with at least one connection to the respective TF are shown).Enrichment (odds ratio ‐ OR) of NFKB1, IRF8, IRF1 and IRF2 target genes identified in cell‐type specific knockouts (K/O, rows) in the matching macrophage, AML and T‐cell eGRN regulons (columns). Numbers in cells indicate: (# genes in regulon and down in TF K/O)/(# genes in regulon). Asterisks indicate significance using Fisher's exact test; test set: all protein‐coding genes; categories: gene in regulon, gene down in TF K/O (NS: non‐significant, *P‐adj. < 0.05, ****: < 0.001). White squares indicate empty regulons.Enrichment of T‐cell and macrophage‐specific FANTOM5 CAGE enhancers among the T‐cell and macrophage eGRN peaks. The numbers inside the tiles are BH‐adjusted *P*‐values based on Fisher's exact test; test set: all peaks in the respective cell types (102,141 and 248,844 for T‐cells and macrophage eGRNs, respectively); categories: peak in eGRN, peak overlap with CAGE enhancer. The macrophage eGRN is the union between the infected, naive and primed eGRNs.

GRaNPA requires differential RNA expression data for a perturbation in the cell type for which the GRN was constructed, and that is independent from the data used to generate the GRN. It then trains a random forest regression model to predict a differential expression value per gene, based on the TF‐target gene connections from the GRN in a 10‐fold cross validation setting (see Materials and Methods; Fig 2A), using *R* ^2^, area under the precision‐recall, and receiver operating curves (AUPRC and AUROC) to measure performance. To ensure the prediction is specific to the real GRN, it also trains a separate random forest model based on a permuted GRN, constructed from the same TFs and genes by permuting the edges (thus conserving the degree distribution of the real GRN). A good performance of the permuted GRN indicates that even unspecific TF‐gene connections can predict differential expression, invalidating the real network's specificity. Lastly, to assess overfitting, GRaNPA trains the same permuted network on completely random differential expression data (uniform distribution between −10 and 10; see Materials and Methods). If GRaNPA performs well on this random data, the model is likely overfitting. Notably, GRaNPA can be applied to assess any GRN that contains TF‐gene connections and may be used to benchmark GRNs constructed using various methods (see below). Furthermore, given a predictive GRN and specific differential expression data, GRaNPA estimates the importance of each TF towards the prediction, which provides candidate driver TFs for a specific expression response. The calculation of TF importance is based on the built‐in *importance* function in the R package *ranger* that quantifies importance of features in random forest models based on how much their exclusion affects prediction accuracy.

In short, the GRaNPA strategy is based on the following steps:

- Obtain differential expression data for the cell type matching the GRN.
- For each cell type, train a random forest regression model (10‐fold cross‐validation) to predict a differential expression value per gene based on TF‐gene links from the GRN.
- Compare the performance of models learned on real and permuted TF‐gene links, and TF‐gene links from other cell types (cross‐validation *R* ^2^).
- Identify important TFs for the given differential expression response.

GRaNPA is implemented as a user‐friendly R‐package (https://git.embl.de/grp‐zaugg/GRaNPA) and documentation is available at (https://grp‐zaugg.embl‐community.io/GRaNPA/). Limitations of GRaNPA are outlined in Box 2.

Box 2 **Limitations of GRaNPA**

- GRaNPA is based on the assumption that differential gene expression, which is always based on steady‐state RNA expression levels, is explained solely by the action of TFs. This is a simplification and other processes, such as RNA stability, also affect RNA expression levels.
- The performance values from GRaNPA are often low, even if they are better than those for permuted networks, suggesting that the GRNs are not picking up all the signal in the data. Adding gene‐specific features e.g. from (Sigalova *et al*, 2020) may substantially improve performance if desired.
- GRaNPA cannot resolve cooperative TF binding.
- GRaNPA fails for datasets in which only a small number of differentially expressed genes overlap with the tested GRN.
- TFs with few connections in the GRN are less likely to be identified as important TFs with GRaNPA, simply because they do not affect many genes.

### GRaNPA evaluation of the macrophage eGRNs

To evaluate the predictive power of the macrophage eGRNs and identify the TFs driving a specific expression response, we obtained RNA‐seq data for naive and Salmonella‐infected macrophages from (Alasoo *et al*, 2018), and calculated the differential expression using DESeq2 (Love *et al*, 2014; Materials and Methods). For evaluations, we excluded samples that were used for the eGRN reconstruction.

The three macrophage eGRNs performed well with GRaNPA, predicting differential expression values (random forest regression) with *R* ^2^ of 0.15–0.25 (Fig 2B and C, Appendix Figs S10 and S11) and direction of change (classification) with AUPRC of 0.71–0.88 and AUROC of 0.65–75 (Appendix Figs S12 and S13). The performance of the corresponding permuted networks was significantly lower (*t*‐test *P*‐value < 1e‐6 for all; Fig 2B and C, Appendix Figs S11–S13). Notably, the eGRN for primed‐infected macrophages that we excluded above due to failed ChIP‐seq validation (Appendix Fig S2) was unable to predict any differential expression (Appendix Figs S10–S13), which highlights the concordance of GRaNPA evaluation with molecular evidence. The significant difference between the permuted and the actual networks shows that the eGRNs indeed capture biologically relevant links between TFs and genes.

### eGRNs built from single cell types show cell‐type‐specific predictions

We next assessed the cell‐type specificity of GRaNIE‐inferred eGRNs. To this end, we obtained data sets in different cell types with matched RNA and chromatin accessibility data for primary human CD4^+^ T‐cells (Freimer *et al*, 2022) and from AML (Garg *et al*, 2019) and (He *et al*, 2022). We ran GRaNIE using the same parameters as described above and obtained additional eGRNs for primary CD4^+^ T‐cells (Dataset EV5) and AML (Dataset EV6).

To assess their cell‐type‐specific prediction power, we ran GRaNPA on the naive macrophage, T‐cell, and AML eGRNs, and compared their performance to predict differential expression in each of the three cell types. Specifically, we quantified differential expression between resting and lipopolysaccharide (LPS) stimulated follicular CD4^+^ T‐cells (data from Calderon *et al*, 2019), between two subtypes of AML (GPR56‐high vs. GPR56‐low; data from Garg *et al*, 2019), and between naive and Salmonella‐infected macrophages (data from Alasoo *et al*, 2018). We found that the eGRN that matches the respective cell type led to the best prediction (Fig 2D). While T‐cells and macrophages were only predictive in their own cell type, the AML eGRN was to a smaller extent also predictive for the macrophage response. Since AML cells and macrophages are both from the myeloid lineage, this could indicate some shared regulatory architecture between them. Notably, we found that the *R* ^2^ values can be boosted by adding gene specific features, such as expression variation across individuals, in line with our previous work (Sigalova *et al*, 2020) (Appendix Fig S14). We are primarily interested in evaluating TF‐gene links and eGRN cell‐type specificity, so GRaNPA does not use these gene‐specific features by default.

Using the TF‐importance estimation implemented in GRaNPA, we observed that among the top five important TFs, most are unique for one cell type (Fig 2E) with the exceptions of IRF8, which was important in AML and macrophages, and SP4, important for AML and T‐cells. Notably, the IRF8 regulons in AML and macrophages had only 22 genes (and no single enhancer) in common, while each cell‐type specific regulon included hundreds of nonoverlapping genes (Fig 2F). Similarly, the SP4 regulons of T‐cells and AML were almost mutually exclusive. This suggests a highly cell‐type‐specific regulon composition of IRF8 and SP4.

As an orthogonal validation of the cell‐type specificity of the TF regulons from GRaNIE eGRNs, we compared the regulons with differential expression data upon TF knockout (K/O) in the same cell type. We obtained data for one or two of the top five important TFs in each cell type: *NFKB1* in macrophages (Somma *et al*, 2021), *IRF8* in AML (Liss *et al*, 2021) and *IRF1* and *IRF2* in T‐cells (Freimer *et al*, 2022). The genes downregulated upon TF K/O were significantly enriched in the TF regulons of the respective cell types (Fig 2G). Notably, genes downregulated upon *IRF8* K/O in AML were specifically enriched for the IRF8 regulon in AML, despite the fact that IRF8 also has a large regulon and is an important TF in macrophages (Fig 2E). This suggests that the cell‐type specificity of the GRaNPA predictions is not only dependent on distinct sets of TFs driving the response, but also on the genes the TF regulates in that cell type, highlighting the importance of cell type‐specific eGRNs.

To validate the cell‐type specificity of enhancers in GRaNIE, we obtained cell‐type‐specific enhancer maps from FANTOM5 using CAGE data for T‐cells and macrophages (Andersson *et al*, 2014). Quantifying their overlap with enhancers from T‐cell and macrophage eGRNs revealed a stronger significant enrichment among the enhancers from the same cell type as compared with opposite cell types (Fig 2H).

The eGRNs connect TFs to genes through active regulatory regions, comprising both promoters and enhancers. The predictive evaluation set‐up allowed us to compare the relative importance of promoter (i.e. < 10 kb from TSS) and enhancer links (> 10 kb from TSS) in different eGRNs. To do so, we divided the gene‐peak pairs into 10 groups based on their distance to the TSS and ran GRaNPA for each group separately. The promoter‐only eGRNs from infected and primed macrophages showed limited or no predictive power (Appendix Fig S15). This highlights the importance of enhancers and is in line with a recent study that demonstrated the importance of considering enhancers for predicting the cell‐fate potential of TFs (Xu *et al*, 2021).

### Application of GRaNPA to compare GRaNIE eGRNs with other GRN methods

Notably, GRaNPA is applicable to assess any type of bipartite TF‐gene network and can be used more generally to assess the utility of a GRN for understanding cellular response to specific perturbations. Here, we used it to evaluate the performance of several previously published TF‐gene GRNs that draw links between TFs and genes based on different approaches: DoRothEA, which uses manual curation combined with a data‐driven approach including co‐expression to draw TF‐gene links (Garcia‐Alonso *et al*, 2019; Holland *et al*, 2020a, 2020b), ChEA3, which uses ChIP‐seq experiments from ENCODE, ReMap, or literature to draw TF‐gene links (Keenan *et al*, 2019), RegNet, a curated network integrating TFs and miRNAs (Liu *et al*, 2015), and TRRUST, which is a curation of TF‐gene links based on PubMed indexed articles (Han *et al*, 2018). We also included an enhancer‐based GRN inferred with ANANSE (Xu *et al*, 2021) from macrophage data.

**Figure 3:**
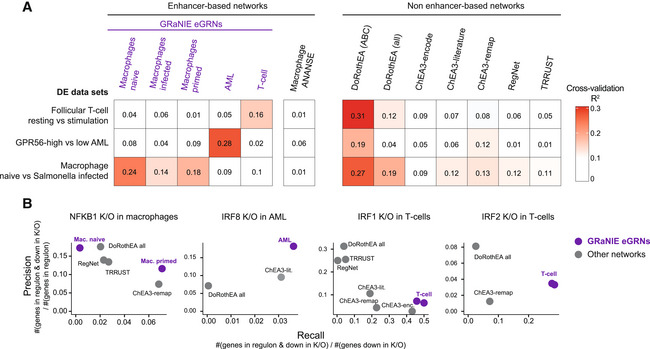
Evaluation of GRaNIE eGRNs and other GRN approaches GRaNPA evaluation of five GRaNIE eGRNs (macrophage naive/primed/infected, AML, and T‐cells), another enhancer‐based eGRN inferred with ANANSE (Xu *et al*, 2021), and publicly available TF‐gene networks based on data curation (DoRothEA ABC and all (Holland *et al*, 2020a)), ChIP‐seq data (ChEA3 encode, literature, and ReMap (Keenan *et al*, 2019)), manual curation (TRRUST (Han *et al*, 2018) and REGNET (Liu *et al*, 2015)). GRNs are evaluated by GRaNPA for their performance in predicting the differential expression of resting versus stimulated follicular T‐cells, GPR56 high versus low AML, and naive versus Salmonella‐infected macrophages. Numbers in squares indicate *R* ^2^ values.Precision‐Recall evaluation of the NFKB1, IRF8, IRF1 and IRF2 regulon from the networks in (A) for identifying genes down‐regulated upon K/O of the respective TF. For GRaNIE eGRNs (purple), the performance of cell‐type matching networks is shown, other networks are the same across all analyses.

The cell‐type‐matched GRaNIE eGRNs and DoRothEA ABC showed good prediction for all datasets tested. The TRRUST, RegNet and ChEA3 networks showed slight predictive power for macrophages, while the only other enhancer‐based network ANANSE showed very poor performance across all cell types (Fig 3A). Thus, GRaNIE networks outperformed most other networks and was on par with the highly curated DoRothEA.

To further compare cell‐type specificity, we assessed the overlap between the TF‐regulons identified in the networks with reasonable predictive power and the genes downregulated upon K/O of the same TF (data introduced in Fig 2G). Overall, the cell‐type‐matched GRaNIE eGRNs outperformed all other networks in terms of recall (Fig 3B). Of note, the absolute recall was rather small, likely owing to the fact that TF K/O induces many indirect downstream effects that are not captured by the direct mechanistic links of eGRNs. GRaNIE also outperformed all other networks in terms of precision in AML. While DoRoThEA achieved the highest precision for *IRF1* and *IRF2* K/O in T‐cells, the recall was smaller. Overall, this cell‐type‐specific TF K/O evaluation highlights the importance of unbiased and cell‐type‐specific eGRNs.

### Macrophage eGRNs reveal distinct set of TFs driving response to different types of infection

GRaNIE and GRaNPA can also provide biological insights. Specifically, we employed them for studying different types of pro‐inflammatory M1‐like responses of macrophages to bacterial infections as well as the anti‐inflammatory M2‐like response of breast cancer associated macrophages. We obtained data from previously published studies (Table 2) that measured the expression response of macrophages infected with *Mycobacterium Tuberculosis* (MTB) (Giraud‐Gatineau *et al*, 2020), *Listeria monocytogenes* (Pai *et al*, 2016), *Salmonella Typhimurium* (Pai *et al*, 2016; Alasoo *et al*, 2018), stimulation with IFN‐γ (Alasoo *et al*, 2018) and a study that compared tumour associated macrophages with tissue‐resident macrophages from breast cancer tissue (Cassetta *et al*, 2019).

**Table 2:** Differential expression experiments for the different infection settings.

Cell types	Treatment	Comparison	Reference
Monocyte‐derived macrophages from healthy donors	Listeria monocytogenes	Uninfected versus 2 h post infection	Pai *et al* (2016)
	Salmonella Typhimurium		
	Mycobacterium Tuberculosis strain resistant to BDQ treatment	Uninfected versus 18 h post infection	Giraud‐Gatineau *et al* (2020)
		Uninfected versus 36 h post infection	
iPSC‐derived macrophages	Salmonella typhimurium	Uninfected versus 5 h post infection	Alasoo *et al* (2018)
		18 h IFN‐γ‐primed versus 18 h IFN‐γ‐primed +5 h post infection	
	Interferon‐gamma stimulation	Naive versus 18 h IFN‐γ treatment	
Tumour‐associated and tissue resident macrophages from human breast tissue	Tumour versus tissue‐resident	Tumour versus respective tissue resident macrophages	Cassetta *et al* (2019)

Differential expression summary for the different infection datasets/cell types, their treatments and the comparisons used for the differential expression analyses.

**Figure 4:**
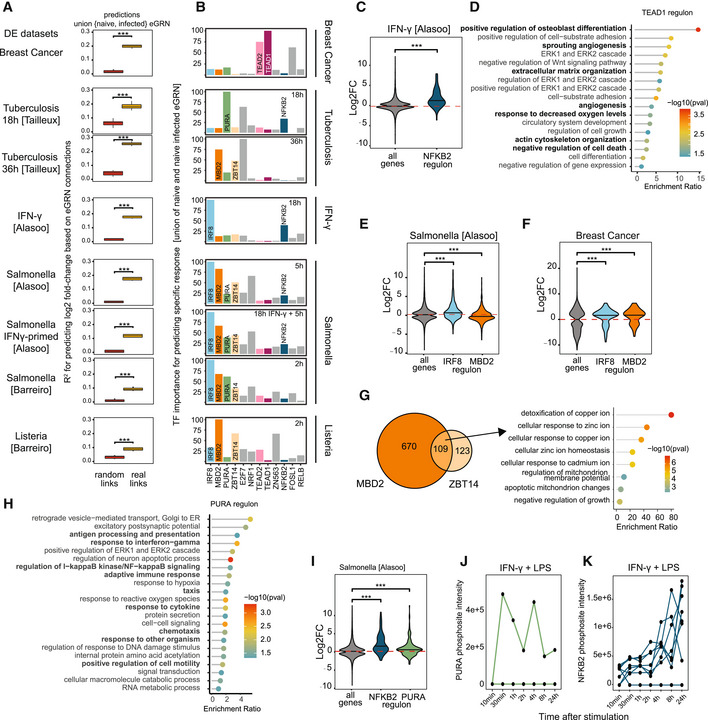
Application of GRaNIE and GRaNPA to investigate macrophage biology AGRaNPA evaluation of the union of the naive and infected macrophage eGRNs (naive+infected eGRN; real links) and the corresponding permuted control network (random links) across eight experimental settings of macrophage perturbations. Distributions of *R* ^2^ from distinct random forest runs (*n* = 10) are shown as boxplots and two sided *t*‐tests were performed to compare GRaNPA performance between the permuted and real networks (***P < 0.001). Boxplots: central band: 50% quantile, box: interquartile range (25–75%); whiskers: max/min are 1.5 IQR above/below the box.BTF importance profiles for each of the eight infection settings from (A). The top 5 most predictive TFs in any of the settings are displayed. TFs discussed in the text are individually labelled and coloured.CDistribution of log2 fold‐changes for genes in the NFKB2 regulons from the naive+infected eGRN (*n* = 85; dark blue) are shown for IFN‐γ stimulation versus naive macrophages alongside the response of all genes (*n* = 2,976; grey). Central band of the violin plot: median.DGO enrichment of the TEAD1 regulon.E, FDistribution of log2 fold‐changes of Salmonella infection versus naive macrophages (E) and for breast‐cancer associated macrophages (F) are shown for genes in the IRF8 (*n* = 830; blue) and MBD2 (*n* = 779; orange) regulons alongside the response of all genes (*n* = 2,976; grey). Central band of the violin plot: median.GThe overlap between the MBD2 and ZBT14 regulons are shown as Venn Diagram (left). Enriched GO terms for the genes in the intersection are shown as a lollipop plot (right).HGO enrichment of the PURA regulon.IDistribution of log2 fold‐changes of Salmonella infection versus naive macrophages for genes in the NFKB2 (*n* = 85; blue) and PURA (*n* = 258; green) regulon alongside the response of all genes (*n* = 2,976; grey). Central band of the violin plot: median.J, KNormalised mass spectrometry intensity values (*y*‐axis) for phosphosites detected on PURA (green, J) and NFKB2 (blue, K) in macrophages cultured in the presence of M1 polarising stimuli (IFN‐γ and LPS) for indicated time points (*x*‐axis). Lines show individual phosphosites detected on each respective TF. Data information: Two‐sided *t*‐test was used to determine statistical significance in (C, E, F, and I); data points correspond to genes in tested regulons (numbers given in panels). ***P‐value < 0.001.

To understand how macrophages respond to these distinct perturbations, we employed GRaNPA using the union of the naive and infected eGRNs (Appendix Fig S16; Dataset EV4), which showed good predictions for all conditions (Fig 4A), and determined the important TFs for each response. One of the well‐understood responses of macrophages is the IFN‐γ‐mediated activation of the NFKB family of TFs (Medzhitov & Horng, 2009). In line with this, we find NFKB2 as one of the most important TFs upon IFN‐γ stimulation (Fig 4B). The NFKB2 regulon was enriched for GO terms related to chemokine signalling and taxis (Appendix Fig S17) and strongly upregulated in response to IFN‐γ (Fig 4C). This demonstrates the ability of GRaNPA to identify biologically meaningful TFs. To assess the robustness of GRaNPA, we compared the TF importance predictions across two independent data sets from Salmonella‐infected macrophages, which revealed very similar profiles despite differences in the experimental set‐up (Fig 4B; iPSC‐derived vs. monocyte‐derived macrophages) and time points (5h and 2h post infection, respectively), thus highlighting the robustness of GRaNPA and the biological congruence between the experiments.

In contrast, across conditions, TF‐importance profiles were highly variable (Fig 4B), likely reflecting different roles of macrophages (M1 vs. M2) and their variable defence mechanisms triggered by the pathogens (Leseigneur *et al*, 2020). Breast‐cancer associated macrophages showed the most distinct profile with TEAD1/TEAD2 as important TFs. GO analysis of the TEAD1/2 regulon revealed a strong enrichment for angiogenesis, osteoblast differentiation and ERK signalling among others (Fig 4D), in line with a more M2‐like phenotype (Corliss *et al*, 2016; Chen *et al*, 2020).

The most important TF for predicting the response to Salmonella infection was IRF8, followed by MBD2 and ZBT14 (Fig 4B). IRF8 is a known pro‐inflammatory interferon response factor, associated with the pro‐inflammatory (M1) polarisation of macrophages (Chistiakov *et al*, 2018), which we confirmed in our data using gene set enrichment analysis (GSEA) of the IRF8 regulon (Appendix Fig S18). Less is known about MBD2 and ZBT14 in macrophages, although MBD2 has been linked to intestinal inflammation in mice (Jones *et al*, 2020) and with an M2 macrophage programme in pulmonary fibrosis (Wang *et al*, 2021). In line with this, the MBD2 regulon was downregulated in response to infection (Fig 4E, Appendix Fig S13) but upregulated in breast cancer‐associated macrophages (Fig 4F), showing the opposite pattern to the IRF8 regulon (Fig 4E and F). We further find an enrichment of the M2 gene set among the MBD2 regulon in breast cancer associated macrophages (Appendix Fig S18). The MBD2 and ZBT14 regulons show significant overlap (Fig 4F, *P* = 3.3e‐13, hypergeometric test) and genes jointly regulated by them are enriched for terms related to response to metal ions (Fig 4G). The use of zinc and copper ions in macrophage defence strategies is well‐documented (Festa & Thiele, 2012; Stafford *et al*, 2013). Given that ZBT14 and MBD2 are important for predicting response to pathogens, but not to IFN‐γ stimulation (Fig 4B), we speculate that ZBT14 and MBD2 may jointly induce a macrophage‐intrinsic mechanism to counteract toxic metal ions, potentially aimed at overcoming the toxic effects of its own weapons.

### GRaNPA identifies PURA as putative proinflammatory TF in macrophages

Among the TFs that are less well known for their role in macrophages, we find PURA for many of the infection settings. In line with a pro‐inflammatory role of PURA, we found GO terms associated with chemotaxis and IFN‐γ response enriched among genes in its regulon (Fig 4H). GSEA found the M1 gene set significantly enriched among the PURA‐regulated differentially expressed genes upon Salmonella infection (Appendix Fig S18). Furthermore, the expression of genes in the PURA regulon were upregulated upon Salmonella infection to a similar extent as the genes in the NFKB2 regulon, which is a known pro‐inflammatory TF (Fig 4I).

To follow‐up on a potential role of PURA in M1 polarisation, we obtained phosphoproteomics data that were collected upon stimulating macrophages with LPS and IFN‐γ towards the M1 phenotype (He *et al*, 2021). This revealed a specific increase in phosphorylation of Thr187 upon LPS/IFN‐γ stimulation (Fig 4J), following a similar pattern of increasing phosphorylation over time as for phosphosites on NFKB2 (Fig 4K). Notably, this stimulation‐induced phosphosite in PURA is located in the Purα repeats region, which is implicated in DNA binding and crucial for PURA function (Weber *et al*, 2016). Phosphorylation of DNA‐binding regions has been associated with activation of other TFs (Hirata *et al*, 1993), suggesting that activation of PURA is perhaps important for M1 polarisation, providing further evidence for its role in macrophages' pro‐inflammatory response.

Overall, these results highlight the use of GRaNPA in conjunction with cell‐type‐specific eGRNs for investigating the biological functions that are regulated by a TF in a specific cell type.

### Macrophage‐specific eGRNs are enriched in fine‐mapped GWAS variants and immune‐related traits

The strength of the eGRN framework is that we can specifically investigate the role of gene regulatory elements such as enhancers, which are enriched for disease‐associated genetic variants (Claringbould & Zaugg, 2021). We therefore sought to explore the macrophage eGRNs to learn about gene regulatory mechanisms underlying associations of genetic variants with common complex traits and diseases.

**Figure 5:**
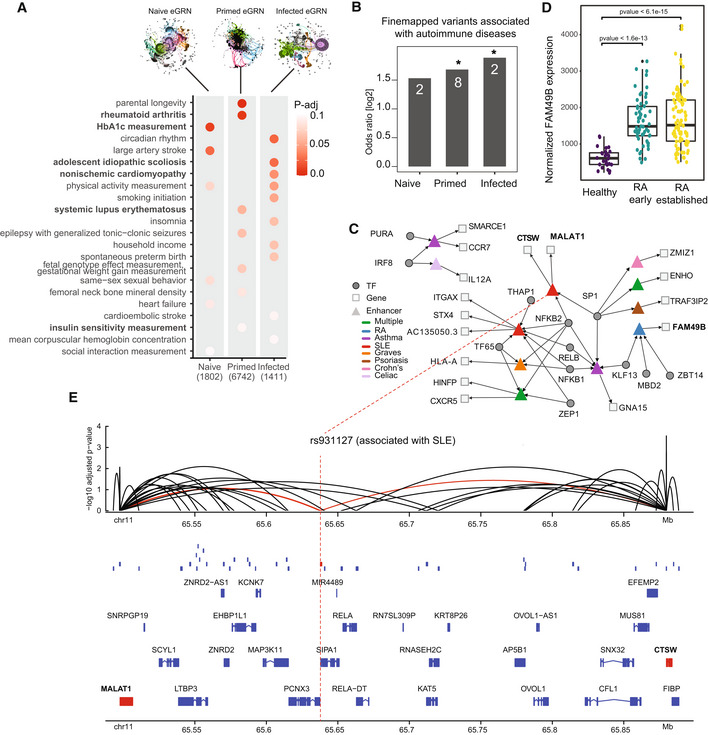
Application of GRaNIE for investigating trait‐associated SNPs Heritability enrichment is shown for the naive, primed and infected macrophage eGRNs. The *P*‐value is adjusted within each trait.The enrichment of fine‐mapped GWAS SNPs within the naive, primed, and infected eGRNs is shown as odds ratios; *P‐value < 0.05 (Fisher's exact test, test set: all ATAC‐seq peaks in macrophages – 296,220; categories: peak in eGRN, peak overlap with finemapped SNP); *n*: number of finemapped SNPs.The tripartite TF‐enhancer‐gene network involving all fine‐mapped GWAS variants for autoimmune diseases.Normalised expression level of *FAM49B* is shown as a boxplot for synovial tissue from healthy controls (*n* = 28) and patients suffering from early (*n* = 57; green) and established (*n* = 95; yellow) rheumatoid arthritis (RA). Data from (Guo *et al*, 2017). Boxplots: central band: 50% quantile, box: interquartile range (25–75%); whiskers: max/min are 1.5 IQR above/below the box. Black dots indicate outliers. Adjusted *P*‐values were calculated according to the Wald test implemented in DESeq2; replicates are individual donors.The genomic context of the fine‐mapped, SLE‐associated variant rs931127 in an ATAC‐seq peak (red box) as gene tracks, including other peaks present in the infected macrophage eGRN (blue boxes), and peak‐gene links from the infected macrophage eGRN (arcs). Genes targeted by the peak overlapping with rs931127 (red) are coloured in red.

First, we tested whether the peak regions specific to the three macrophage eGRNs that GRaNPA identified as predictive in at least one infection setting (naive, primed, infected) were enriched in heritability for 442 GWAS traits (Dataset EV9). We applied stratified linkage disequilibrium score regression (S‐LDSC; Finucane *et al*, 2015) and compared the eGRN peaks to all peaks identified in macrophages (see Materials and Methods). Notable enrichments include HbA1c measurement (a measure for diabetes severity), large artery stroke and heart failure for the naive eGRN; adolescent idiopathic scoliosis and nonischemic cardiomyopathy for the primed eGRN and rheumatoid arthritis (RA) and systemic lupus erythematosus (SLE) for the infected eGRN (Fig 5A). SLE and RA are both driven by activated macrophages (Udalova *et al*, 2016; Ma *et al*, 2019) as a result of known (for SLE) or hypothesised (RA) upregulation of IFN‐γ signalling (Harigai *et al*, 2008; Rönnblom & Leonard, 2019; Kato, 2020). Interestingly, we find enriched heritability for these traits in the peaks for the IFN‐γ primed eGRN, but not for either naive or infected eGRNs. Given this association, we also assessed the heritability enrichment of the regulatory elements and genes connected to the TFs that are particularly important for predicting the response of macrophages to IFN‐γ (NFKB1/2, RELB, IRF8). Inflammatory bowel disease (specifically ulcerative colitis) comes out as the top enriched trait (Appendix Fig S19), which is in line with the known role of IFN‐γ in this disease (Andreou *et al*, 2020). Literature evidence for other traits is summarised in Dataset EV10.

Next, we zoomed in to a specific set of fine‐mapped GWAS variants associated with autoimmune diseases with a known link to macrophages. Across all three macrophage eGRNs, we found in total 11 unique fine‐mapped variants that were located in the regulatory regions (2 in the naive, 2 in infected and 8 in the primed eGRN). The infected and primed eGRNs were significantly enriched in fine‐mapped variants (Fig 5B). Investigating the TFs regulating the fine‐mapped autoimmune disease enhancers, we find the known immune response TFs NFKB1/2, RELB and IRF8, but also MBD2, ZBT14 and PURA (Fig 5C), which we identified as important TFs for predicting response to infection. One of the enhancers overlapping with an RA‐associated SNP, regulated by MBD2 and ZBT14, is linked to *FAM49B as a target gene*. Inspection of *FAM49B* expression in synovial tissue in a cohort of RA patients compared with healthy controls (Guo *et al*, 2017) revealed a misregulation upon disease onset, providing additional evidence that *FAM49B* is indeed the gene targeted by the fine‐mapped SNP (Fig 5D).

One of the fine‐mapped SNPs for SLE, rs9893132, is located in an enhancer regulated by SP1 and NFKB2 and linked to the noncoding RNA *MALAT1* and the gene cathepsin W (*CTSW*) in the primed macrophage eGRN. Both genes are over 50 kb from the fine‐mapped SNP and there are several other genes in the locus that are not linked to the enhancer in question (Fig 5E). *MALAT1* has been implicated in SLE through several studies (reviewed in Zhao *et al*, 2018), suggesting that rs9893132 may target *MALAT1*. While *CTSW* expression in lymphocytes has been linked to autoimmune diseases (e.g. Buhling *et al*, 2002), its role in macrophages is much less studied. Yet, *CTSW* knockdown in macrophages reportedly increased *Mycobacterium tuberculosis* survival in macrophages (Pires *et al*, 2016), suggesting it does play an important function in the pro‐inflammatory macrophage response. Overall, the macrophage eGRNs provide the putative target genes of 11 fine‐mapped GWAS loci, often linking to genes that are over 50 kb away from the SNP (Table 3; Appendix Fig S20).

**Table 3:** Predicted target genes of fine‐mapped GWAS autoimmune variants using macrophage eGRNs.

Peak	TF	Gene name	FM.gwas	rsid	Disease	Network
chr8:129939305‐129940672	KLF13.0.D, MBD2.0.B, ZBT14.0.C	FAM49B	chr8:129939865	rs11785995	Rheumatoid arthritis	Naive
chr17:40598595‐40599644	PURA.0.D	SMARCE1	chr17:40598769	rs9893132	Asthma	Naive
chr19:3135922‐3136231	KLF13.0.D, NFKB1.1.B, NFKB2.0.B, RELB.0.C, SP1.0.A, SP1.1.A	GNA15	chr19:3136093	rs117552144	Asthma	Primed
chr16:31265059‐31265802	NFKB1.1.B, NFKB2.0.B, RELB.0.C, TF65.0.A, THAP1.0.C, ZEP1.0.D	STX4, AC135050.3, ITGAX	chr16:31265490	rs1143679	Systemic lupus erythematosus	Primed
chr6:30006131‐30007036	NFKB1.1.B, NFKB2.0.B, TF65.0.A	HLA‐A	chr6:30006148	rs4313034	Graves' disease	Primed
chr11:65637802‐65638765	NFKB2.0.B, SP1.0.A, SP1.1.A	MALAT1, CTSW	chr11:65637829	rs931127	Systemic lupus erythematosus	Primed
chr11:118883323‐118883647	NFKB2.0.B, TF65.0.A, ZEP1.0.D	CXCR5, HINFP	chr11:118883644	rs630923	Multiple sclerosis, Inflammatory bowel disease, Crohn's disease	Primed
chr6:111605185‐111606373	SP1.0.A	TRAF3IP2	chr6:111605706	rs7769061	Psoriasis	Primed
chr9:34709959‐34710335	SP1.0.A, SP1.1.A	ENHO	chr9:34710263	rs2812378	Rheumatoid arthritis, Celiac disease	Primed
chr10:79285352‐79285717	SP1.1.A	ZMIZ1	chr10:79285450	rs1250569	Crohn's disease	Primed
chr10:79285352‐79285717	SP1.1.A	ZMIZ1	chr10:79285523	rs1250568	Celiac disease	Primed
chr17:40598595‐40599644	IRF8.0.B	CCR7	chr17:40598769	rs9893132	Asthma	Infected
chr3:159929439‐159930124	IRF8.0.B	IL12A	chr3:159929885	rs17753641	Celiac disease	Infected

Fine‐mapped GWAS variants for autoimmune diseases generated using probabilistic identification of causal SNPs (PICS; see Materials and Methods) for *hg38* build overlapping with the peaks of macrophage eGRNs. TF names refer to specific motifs, some TFs have multiple motifs and thus occur multiple times.

## Discussion

Phenotypic variation across individuals has two major sources: genetic variation and external influences that can be long‐lived (epigenetics) or short‐lived (signalling). Both can lead to variation in molecular phenotypes that impact on complex traits. Thus, to understand mechanisms underlying phenotypic variation, including disease phenotypes, it is crucial to study the interplay between genetic variants, epigenetic marks and extrinsic cellular signalling. Here, we present GRaNIE and GRaNPA, a tool‐suite that provides a framework for jointly analysing these layers and investigating their biological relevance.

GRaNIE is a flexible and user‐friendly R/Bioconductor package for building enhancer‐based GRNs. It requires RNA‐Seq and open chromatin data such as ATAC‐Seq or ChIP‐Seq for histone modifications (e.g. H3K27ac) across a range of samples (mostly tested in a cohort of at least 10–15 individuals), along with TFBS data (that can either be obtained from the package or provided by the user) to generate cell‐type‐specific eGRNs. It provides a range of quality control plots and functionalities for downstream analyses such as identification of communities within the network, and GO enrichment analyses. A dedicated website accompanies the package and is automatically updated whenever a new package version becomes available.

GRaNPA is an independent R package for evaluating the biological relevance (i.e. predictive power) of any TF‐gene network. It requires a bipartite TF‐gene network and genome‐wide differential expression values as input, and assesses the network's predictive power. In addition, it quantifies the importance of each TF for driving a specific differential expression response. Notably, the prediction performance of GRaNPA can be improved by adding gene‐specific features, for example those shown in (Sigalova *et al*, 2020); however, this would not help in the assessment of GRNs and is therefore not the main purpose of this study. One attractive use case of GRaNPA is that it can quantitatively compare the performance of different GRNs for predicting a perturbation of interest. It can thus help select the best‐suited network for a given dataset without the need for a “ground truth” network to evaluate their edges and connectivities.

Compared to most of the available GRN reconstruction approaches, GRaNIE infers enhancer‐based regulatory networks that only captures TF‐gene links mediated by enhancers. This has several advantages: first, we showed that for macrophages, parts of their expression response to infection could only be predicted when using enhancer connections. Second, including enhancers in TF‐driven GRNs allows the investigation of mechanisms underlying GWAS traits that are driven by specific TFs, and facilitates interpretation of (fine‐mapped) trait‐associated SNPs. Third, since enhancers tend to be highly cell‐type specific (Roadmap Epigenomics Consortium *et al*, 2015), eGRNs are likely more cell‐type specific than TF‐gene networks. Finally, by requiring a correlation between the expression level of a TF and the accessibility of the peak, in addition to the motif presence, GRaNIE circumvents the inherent limitation of TF‐binding site predictions, which cannot distinguish between TFs with similar binding motifs (Zeitlinger, 2020). This will exclude many TF‐enhancer links that have the TF motif yet show no correlation with the TF expression level and are thus likely not bound by that TF in the given cell type. GRaNIE bears conceptual similarity with a method published previously (Marbach *et al*, 2016); however, the data of this work are not available anymore, and the software is neither maintained nor can be downloaded/used.

Enhancer‐based gene regulatory networks consist of TFs and their respective downstream enhancer/promoter and gene targets, which means we can zoom into network communities that capture specific pathways or functions. For example, we showed that when we divide the network into communities, each community is enriched in distinct TF‐driven biological processes. The modularity of the eGRN also showed that NFKB1, NFKB2, RELB and IRF8, the TFs important for predicting the macrophage response to IFN‐γ priming, and their connected regulatory elements and genes were specifically enriched for heritability of GWAS traits that are commonly linked to IFN‐γ signalling. In contrast to other approaches for interpreting trait‐associated variants that are solely based on epigenetics such as the activity by contact model (Nasser *et al*, 2021), purely based on genetics, such as eQTLs (Võsa *et al*, 2021) or variable chromatin domains (Waszak *et al*, 2015), eGRNs by GRaNIE capture TF‐peak‐gene links based on variation due to genetic, epigenetic, or TF‐activity differences across individuals, thus integrating these three layers in one framework. In sum, eGRNs can be used to identify the target genes of individual TFs, to pinpoint the cell‐type‐specific regulatory regions that connect to a TF, and to investigate genetic variation in the tripartite TF‐regulatory element‐gene graphs.

Comparing eGRNs across cell types revealed that for some TFs (e.g. IRF8), the regulons are highly cell‐type specific. Cell‐type‐specific TF functions may be driven by different co‐binding TF partners depending on the cell type. Indeed, in our previous work, we found that TFs regulate distinct biological processes depending on their co‐binding partners (Bunina *et al*, 2020; Ibarra *et al*, 2020). An alternative explanation for cell‐type‐specific regulons is that different cell types may differ in their chromatin potential (Ma *et al*, 2020).

Among the notable observations from applying GRaNIE and GRaNPA to study the gene expression response in macrophages was that some TFs, including MBD2, were specifically important only for predicting response to bacterial infection, and not for IFN‐γ stimulation. Since IRF transcriptional programs are generally more related to a virus response, MBD2 may be required for the bacterial‐specific response, indicating that we can use these networks to identify TFs important for responses to different types of pathogens. The observation that GRaNPA identified distinct sets of important TFs for the different responses may reflect that macrophages use several strategies to fight infections, including phagocytosis followed by degradation mechanisms, starvation of pathogens, and recruiting other players in the immune system (Leseigneur *et al*, 2020). Another observation is that three TFs important for predicting the response to infection but not to IFN‐γ, are known to bind methylated DNA: MBD2 (Hainer *et al*, 2016), MECP2 (Lewis *et al*, 1992) and NRF1 (Domcke *et al*, 2015). Recent reports provide evidence for a pathogen‐induced global DNA methylation alteration (Qin *et al*, 2021) downstream of NFKB‐signalling, and it was shown that MBD2 inhibits IFN‐γ by selectively binding to methylated regions in the Stat1 promoter in other cell types (Yue *et al*, 2021). Our results are consistent with a pathogen‐response mechanism that is partially mediated by DNA methylation, which may modulate the impact of DNA‐methylation sensitive TFs and demonstrates the level of novel biological insights that can be gained with GRaNIE and GRaNPA.

## Materials and Methods

### Reagents and Tools table

**Table:** 

Reagent/Resource	Reference or source	Identifier or catalogue number
**Software**		
GRaNIE R package	https://bioconductor.org/packages/GRaNIE/	
GRaNPA R package	https://git.embl.de/grp‐zaugg/GRaNPA	
Gephi 0.10.1	https://gephi.org/	
*DESeq2* R package	https://bioconductor.org/packages/DESeq2/	
*GeneOverlap* R package	https://bioconductor.org/packages/GeneOverlap	
*fgsea R package*	https://bioconductor.org/packages/fgsea	
UCSC liftOver web interface	https://genome.ucsc.edu/cgi‐bin/hgLiftOver	
**External gene regulatory networks**		
*Dorothea*	Garcia‐Alonso *et al* (2019)	
*TRRUST*	Han *et al* (2018)	
*ChEA3*	Keenan *et al* (2019)	
ANANSE	Xu *et al* (2021)	
**Other databases and resources**		
ReMap 2022	Hammal *et al* (2022)	
FANTOM5 Human Enhancer Tracks	https://slidebase.binf.ku.dk/human_enhancers/presets Andersson *et al* (2014).	https://slidebase.binf.ku.dk/human_enhancers/presets/serve/macrophage
eQTL catalogue	https://www.ebi.ac.uk/eqtl/	
LDSC Github repository	https://github.com/bulik/ldsc	
General genomic features dataset	https://alkesgroup.broadinstitute.org/LDSCORE/	
Fine‐mapped GWAS variants for autoimmune diseases	“PICS2‐GWAScat‐2020‐05‐22.txt.gz” from https://pics2.ucsf.edu	

### Methods and Protocols

#### Data sets used in this study

For all data sets, we performed PCA along with metadata inspection in the PCA space to evaluate whether samples should be discarded as outliers. If we did, we give details in the respective paragraph.

##### Expression and chromatin accessibility data for iPSC‐derived macrophages

We used a publicly available data set (ERP020977) for naive and primed macrophages (iPSC‐derived) in two conditions, uninfected and 5‐h infected with Salmonella from (Alasoo *et al*, 2018). In total, we obtained 304 RNA‐seq profiles from 86 different individuals, of which 145 also had ATACseq data available (https://zenodo.org/record/1188300#.X370PXUzaSN). The samples are split into four groups: primed, primed‐infected, naive and naive‐infected for which 41 (43), 31 (55), 42 (42), 31 (55) paired RNA/ATAC (only RNA‐seq) samples were available, respectively. The data also contained metadata and peak coordinates. The paired samples were used to reconstruct the eGRNs with GRaNIE (see below). The unpaired RNA‐seq data were used for evaluation of the eGRNs with GRaNPA (see below).

##### Expression data for macrophages infected with Listeria & Salmonella (GEO accession number: GSE73502)

Pai *et al* (2016) generated expression data on cultured monocytes obtained from PBMCs of healthy donors, for which we downloaded the raw counts data. Matured macrophages were divided into three groups: (i) controls and infected by the (ii) Listeria and (iii) Salmonella bacteria, respectively. We used the RNA‐seq data collected 2 h after infection with Listeria and Salmonella, respectively, for each of the 57 samples.

##### Expression data for macrophages infected with Tuberculosis (GEO accession numbers: GSE133145, GSE143731)

Giraud‐Gatineau *et al* (2020) collected two data sets on the effect of bedaquiline (GSE133145) and five other drugs (GSE143731) treatment for *Mycobacterium tuberculosis* infection in Monocyte‐derived macrophages from healthy donors. The GSE133145 series consists of 16 control and 16 *M. tuberculosis*‐infected samples, which are later divided into four groups: untreated/DMSO treatment (control)/two variants of bedaquiline treatment (0.5 or 5 μg/ml). Differential expression analysis revealed that differences caused by treatment are not substantial, so we considered the treatment as a controlling variable. The GSE143731 series consists of 28 control and 28 *M. tuberculosis*‐infected samples, which are later divided into groups corresponding to the treatment with isoniazid (INH), rifampicin (RIF), ethambutol, pyrazinamide (PZA) or amikacin (AMK), and control group. We considered treatment as a controlling variable for the differential expression analysis.

##### Expression and chromatin accessibility data for CD4 ^+^ T‐cells

Paired RNA‐ and ATAC‐seq data were obtained from (Freimer *et al*, 2022). For RNA‐seq, processed count files were obtained from GSE171737. For ATAC‐seq, raw sequencing files were obtained from GSE171737 and processed and quality‐controlled with an in‐house Snakemake pipeline as previously described (Berest *et al*, 2019).

##### Expression data for resting versus LPS‐stimulated CD4 ^+^ T‐cells (GSE118165)

RNA‐seq was obtained from (Calderon *et al*, 2019), which measured expression in resting and stimulated subsets of CD4^+^ T‐cells. We used the T‐follicular helper cells for differential expression analyses.

##### Expression and chromatin accessibility for AML

We obtained raw RNA‐seq data for 23 AML patients from (Garg *et al*, 2019). Processed and quality‐controlled ATAC‐seq data and peaks for the same patients was obtained from (He *et al*, 2022).

##### Expression data for TF K/Os

We obtained cell‐type‐specific knockout (K/O) data for THP1‐derived macrophages (NFKB1) (Somma *et al*, 2021), the human AML cell line MV4‐11 (IRF8) (Liss *et al*, 2021), and processed differential expression data from primary human CD4^+^ T cells (IRF1 and IRF2) (Freimer *et al*, 2022). For the NFKB1 and IRF8 data sets, raw sequencing files were obtained from GSE162015 and GSE163275, respectively, and data processing and quality control was performed with an in‐house Snakemake pipeline as described previously (Berest *et al*, 2019).

##### Macrophage phosphoproteomics data

Processed quantitative phosphoproteomics data from polarising THP1‐derived macrophages was obtained from (He *et al*, 2022).

#### Differential expression analyses

Differential expression analysis was performed with DESeq2 (Love *et al*, 2014) for all data sets, typically using the contrast between treatment and no treatment or disease and control (see also Table 2). The design formula generally used was therefore “~condition,” unless otherwise stated. Dataset‐specific details of the differential expression analysis datasets are described below. As input for GRaNPA, we generally used shrunken log2 fold‐changes as implemented in *lfcShrink* from DESeq2 with the *apeglm* method (Zhu *et al*, 2019) unless otherwise indicated, even though it is not a strict requirement of GRaNPA to use any particular transformation.

##### iPSC‐derived macrophages infected with Salmonella from

Differential expression was calculated using only the RNA‐seq data that were not used for eGRN reconstruction (Alasoo *et al* 2018). We quantified differential expression for the following contrasts: naive versus infected, naive versus IFN‐γ primed, IFN‐γ primed versus IFN‐γ primed‐infected. The formula used in DESeq2 was “~condition.”

##### Macrophages infected with Listeria and Salmonella from

We analysed the differential expression between control samples, listeria‐infected samples and salmonella‐infected samples separately (Pai *et al* 2016). No samples were removed. Information on the donor was used as a covariate, using the design formula: “~patient + condition.”

##### Macrophages infected with Tuberculosis from

We calculated differential expression between monocyte‐derived macrophages from healthy donors infected with tuberculosis versus control samples (Giraud‐Gatineau *et al* 2020). Data sets GSE133145 and GSE143731 were analysed separately, but with a common design formula. Although there were also multiple treatments, the expression variance was almost exclusively driven by the difference in disease status. We therefore added the treatment as a covariate to the design formula (“~patient + treatment + condition”), but only investigated differential expression between infected macrophages and controls. One control and one infected sample from the GSE143731 series were removed from the analysis, as they were clear outliers in the PCA plot.

##### CD4 ^+^ follicular T‐cells resting versus LPS‐stimulated

We quantified differential expression between CD4‐positive follicular T‐cells in resting versus stimulated condition (Calderon *et al*, 2019). The design formula used in DESeq2 is “~condition.”

##### AML subtypes

Differential expression was calculated using data from (Garg *et al*, 2019) and comparing samples with high leukaemia stem cell burden (GPR56‐high) versus low leukaemia stem cell burden (GPR56‐low samples) based on immunophenotyping as defined in (Garg *et al*, 2019). The design formula was: “~GPR56status.” We did not use shrunken log fold‐changes as input for GRaNPA but we verified that results are qualitatively unchanged when doing so.

##### Tumour‐associated and tissue resident macrophages from human breast tissue

Raw RNA‐sequencing data were obtained from GSE117970 (Cassetta *et al*, 2019), and processed in the same way as described for expression data for TF K/Os. We obtained differentially expressed genes between tissue resident and tumour associated macrophages using the design formula “~condition.”

#### GRaNIE: Construction of eGRNs

The following is needed as input for GRaNIE:

- Raw or prenormalised chromatin accessibility data (e.g. ATAC‐seq, DNase‐Seq or histone modification ChIP‐seq data such as H3K27ac);
- Raw or prenormalised RNA‐seq counts;
- Precompiled lists of TFBS predictions per TF (we provide predictions for human and mouse TFBS that were derived as described in Berest *et al*, 2019); and
- TAD domains (optional).

For all data sets in this study, we used the same default parameters when constructing the eGRNs as described below.

GRaNIE is conceptually based on the procedure described in (Reyes‐Palomares *et al*, 2020) and has the following main steps:

##### (i) Process chromatin accessibility and RNA‐seq data

Both ATAC‐seq and RNA‐seq may be raw counts or prenormalised counts. If raw counts are provided for RNA‐seq, by default we quantile normalised the RNA‐Seq count data in order to minimise the effects of outlier values that may otherwise have a large influence on the resulting correlations. For chromatin accessibility data, we employ a size factor normalisation as implemented in DESeq2 (Love *et al*, 2014). However, the user can define which type of normalisation shall be used for either data. Additional filters for excluding particular chromosomes (e.g. sex chromosomes) or genes/peaks with low counts can optionally be used. The latter is implemented by removing genes/peaks if the average counts across all samples are below a specified threshold (5 by default).

##### (ii) Overlap TF binding sites with ATAC‐Seq peaks

Based on the provided list of putative TFBS per TF (see Berest *et al*, 2019 for details), we overlap all TFBS from all TF with the open chromatin peaks and record for each peak and TF whether at least one putative TFBS is located within the peak. This binary TF‐peak binding matrix is used in subsequent steps.

##### (iii) Identify statistically significant TF ‐ peak connections

To identify statistically significant TF‐peak connections, we implement a cell‐type‐specific data‐driven approach. In brief, we first calculate the Pearson's correlation coefficients between the expression level of each TF and the open chromatin signal of each peak across all samples.

We then use an empirical FDR procedure to identify statistically significant TF‐peak connections. For this, for each TF, we split the peaks into two sets: a foreground set containing the peaks with a predicted TFBS and a corresponding background set consisting of peaks without predicted TFBS based on the TF‐peak binding matrix calculated above. We then discretize the TF‐peak correlation *r* into 40 bins in steps of 0.05 ranging from −1, −0.95, …, 0, …, 1 and calculate a bin‐specific FDR value using two different directions (*positive*: left to right from −1 to 1, *negative*: right to left from 1 to −1). For each bin (correlation threshold) *k*, we calculate the empirical FDR according to the formula ${\text{efdr}}_{k}=\frac{{\mathrm{nfp}}_{k}}{{\mathrm{nfp}}_{k}+{\mathrm{ntp}}_{k}}$, with ${\mathrm{nfp}}_{k}$ and ${\mathrm{ntp}}_{k}$ denoting the total number of TF‐peaks in the background and foreground, respectively, for which r≥k (direction positive) and r<k (direction negative). To make the numbers from foreground and background compatible, we normalise ${\mathrm{nfp}}_{k}$ beforehand by their ratio (i.e. ${\mathrm{nfp}}_{k}={\mathrm{nfp}}_{k}\times \frac{\mathrm{ntp}}{\mathrm{nfp}}$, with ntp and nfp denoting the total number of TF‐peaks in the foreground and background, respectively).

##### (iv) Activator‐repressor TF classification (optional)

Optionally, the TF classification as described in (Berest *et al*, 2019) can be run and is fully integrated in GRaNIE. It produces a classification of TFs into putative activators, repressors or undetermined. Briefly, it compares the distribution of correlations for peaks with putative binding sites (foreground) against all other peaks (i.e. background) and classifies TFs depending on whether the correlations of putative targets are significantly more positive than (activator), more negative than (repressor) or indistinguishable from (undetermined) the background.

##### (v) Identify statistically significant peak‐gene connections

Next, we add peak‐gene connections to our network. We identify highly correlated peak‐gene pairs based on their Pearson's correlation and the associated *P*‐value (using cor.test in R) between the normalised RNA‐seq for the expression of a gene and the corresponding open chromatin peak.

GRaNIE offers two options to decide which peak‐gene pairs to test for correlation: in absence of additional topologically associating domain (TADs) data from Hi‐C or similar approaches it uses a local neighbourhood‐based approach with a custom neighbourhood size (default: 250 kb up‐ and downstream of the peak) to select peak‐gene pairs to test. In the presence of TAD data, only peak‐gene pairs within a TAD are tested. The user has furthermore the choice to specify whether overlapping TADs should be merged or not. We offer two options of where in the gene the overlap with the extended peak may occur: at the 5′ end of the gene (the default) or anywhere in the gene.

GRaNIE also records additional properties for each peak‐gene pair such as their distance as well as gene type & status as annotated by Gencode. By default, only protein‐coding and lincRNA genes are kept in the eGRN, but this can be customised to include other gene types.

#### (vi) Filter GRN connections and calculate peak‐gene FDR

Lastly, we offer a variety of options to combine and filter TF‐peaks and peak‐genes to derive the full GRN for subsequent analyses. For example, both types of connections can be filtered by their FDRs or by their correlation, peak‐gene links can further be filtered by their distance, gene type, and other criteria. By default, we retain only peak‐gene pairs that are positively correlated. After applying all filters for the peak‐gene links, multiple testing adjustment is performed using Benjamini–Hochberg. The default thresholds for TF‐peak and peak‐gene links are FDR < 0.2 and FDR < 0.1, respectively.

Lastly, we provide heatmaps and boxplots that compare the connectivity for the real and random eGRNs.

#### GRaNIE quality controls

The package optionally offers PCA plots for both the RNA‐seq and open chromatin data, and upon availability of additional metadata that can be provided, the PCA data can also be coloured accordingly. This facilitates the detection of batch effects and outlier samples that may introduce unwanted variation.

In addition, we implemented a range of quality controls for the different steps to assess both the number as well as signal‐to‐noise for both TF‐enhancer and enhancer‐gene links.

For assessing TF‐enhancer links, we compare the number of links obtained from the real data to a background set of links that we obtain with randomised data using a twofold randomisation scheme (permuting the TF‐peak overlap matrix and sample labels for the RNA counts) by applying the same methods as described before.

For assessing the true enhancer‐gene links, we similarly construct a set of background links. First, after creating the real table for the peak‐gene pairs that fulfill the user‐specified requirements for being tested for correlation, the peaks are shuffled. Notably, this preserves the degree distribution for both peaks and genes. Second, we shuffle the sample labels for the RNA data.

We then base our quality controls on the assumption that peak accessibility and gene expression are positively correlated. Thus, assessing the amount of signal (i.e. small *P*‐values) of positive versus negative correlations in the real versus the background enhancer‐gene links serves as a proxy for the signal to noise ratio. Specifically, we expect that positive correlations outnumber negative ones for the real enhancer‐gene links, which should not be the case for the background. Lastly, we have a number of additional QC plots that include the enhancer‐gene distance, for which we similarly expect a signal difference for the real but not the background links.

#### GRaNIE: Downstream analyses implemented in the package

The following functionalities are available within the GRaNIE package:

- Descriptive statistics pertaining to the structure of the graph, such as the number of nodes and edges and their types, the distribution of node degrees and the top nodes with regard to degree centrality and eigenvector centrality.
- Community identification, for which the package supports multiple algorithms (louvain, walktrap, leading eigenvector, fast greedy and optimal). The communities can be supplemented with descriptive statistics similar to those previously described, but specific to each individual community.
- Enrichment analyses in three different flavours; a general enrichment analysis for the whole network, a community‐based enrichment analysis, or a TF‐based enrichment analysis.

#### Molecular evaluation of GRaNIE‐inferred TF‐peak links using ChIP‐seq

We obtained macrophage‐specific ChIP‐seq data from ReMap 2022 (Hammal *et al*, 2022) for all TFs that were present in any of the GRaNIE inferred eGRNs (CEBPA, CEBPB, FOS, GABPA, GFI1, IRF8, IRF9, LYL1, MYB, NR1H3, PPARG, RUNX1, STAT1, STAT2, VDR). For these TFs we determined the overlap of GRaNIE inferred TF‐linked peaks (independent of whether they also are linked to a gene) with the respective ChIP‐seq peaks (within 50 bp) and calculated the enrichment over the background set of ATAC‐seq peaks that just contained the TF motif using Fisher's exact test. We excluded two TFs (SPI1 and CTCF) that had more than 10 k connections in GRaNIE at 0.4 or 0.5 FDR thresholds but no connections at lower FDR thresholds.

#### Molecular evaluation of GRaNIE‐inferred peak‐gene links using eQTL data

We downloaded *cis*‐eQTL data from the eQTL catalogue (https://www.ebi.ac.uk/eqtl/, accessed on May 5th 2022), selecting all six data sets with eQTLs in monocytes or macrophages. We combined the eQTLs from all data sets and filtered the associations to have a permutation‐based FDR < 0.3. Only GRN peaks that harbour at least one eQTL SNP in these data sets can be evaluated. For every peak, we overlapped the eQTL SNPs, and counted a peak‐gene link as validated if any eQTL SNP affected the same gene as present in the GRN. To test whether the overlap between peak‐gene links and eQTL is enriched as compared to a random background, we also validated links between the GRN peaks and randomly sampled distance‐matched genes based on 50 kb bins. We repeated the random background sampling 20 times and calculated the odds ratio between validated GRN links and validated background links. We calculated the enrichment of validated links by eQTL overlap for a range of peak‐gene FDR thresholds in each of the four macrophage GRNs. We did not add any extra eQTLs from SNPs that were in high linkage disequilibrium with the eQTLs from the six data sets in our link validation. This means we could have missed some eQTL overlaps, but since we used eQTLs from six different datasets, we did include several variants per peak.

#### Molecular evaluation of GRaNIE‐inferred TF‐peak‐gene links

We obtained the tracks of human enhancers identified using CAGE data from FANTOM5 that were differentially expressed in T‐cells and macrophages (Andersson *et al*, 2014). Track coordinates were translated to GRCh38 using UCSC *liftOver* tool (Hinrichs *et al*, 2006). We quantified the overlap of GRaNIE eGRN enhancers (peaks linked to a TF and a gene) and the respective cell‐type specific (macrophage in Figs 1F and 2H and T‐cell in Fig 2H) CAGE peaks and tested for the association of eGRN peaks (vs. all other peaks within 250 kb of a TSS) and cell‐type‐specific CAGE enhancers using Fisher's exact test.

#### Utility evaluation of GRaNIE‐inferred eGRN regulons using GO‐term specificity

To assess the specificity of the top GO terms enriched for the predictive TFs (Appendix Fig S9A), we randomised the TF‐gene connections and recalculated the enrichment. Manual inspection suggested that the returned terms for the random network are more general and often unrelated to macrophage biology. To quantify the specificity, we plotted the distribution of the number of genes annotated to each returned GO term for the real network, and for five permuted networks (Appendix Fig S9B), following the rationale that more general terms would have more genes associated with them in the database.

#### GRaNPA: Prediction model

For the prediction model we do the following:

- Compute the differential expression resulting from a perturbation between two different conditions, for which we used DESeq2. We define DE(j) as the differential expression value for the j^th^ Gene.
- Define the GRN as a mathematical function:

$$grn\left(gene\text{,}tf\right)=\left\{{}_{0\;otherwise}^{1\;tf\;and\;gene\;connected\;in\;the\;GRN}\right\}$$

Based on this, we construct a matrix X, a relation matrix between genes and TFs where each row is a gene and each column is a TFs. There is a 1 in the cell for a gene and TF if and only if they are connected through the GRN.

iii Running a Random Forest (RF) Regression based on the following formula to predict a differential expression value for each gene *i* based on its connected TFs $X_{i,\mathrm{tf}}$:
$$\mathrm{DE}∼\hat{F}\left(X_{\mathrm{TFs}}\right)$$

Random Forest has been implemented using the “ranger” package in R. To avoid overfitting we use 10‐fold cross validation; no hyper‐parameter tuning on the random forest was performed. We measure performance by assessing the cross‐validation *R* ^2^.

#### GRaNPA: Construction of the permuted network to assess edge‐specificity

To assess the edge‐specificity of our network during the random forest regression, we constructed a permuted control network based on the structure of the actual GRN. It has the same number of edges with the same degree distribution for TFs with only the gene labels permutated so the connection between TFs and genes are randomised. The differential expression values are unchanged. In case we use any weighing method for the edges, the same method will be applied for each edge of the permuted version to recalculate weight if needed.

#### GRaNPA: Random signal generation to assess overfitting

To control for over‐fitting in the random forest regression, we used the real network structure and assigned randomly generated values to genes instead of their differential expression value. The random values are chosen from a uniform distribution with minimum and maximum of −5 and 5, respectively. Any prediction for this network is a consequence of overfitting. The reason we used a uniform distribution is that we want to check overfitting and we do not want the distribution to be like any differential expression distribution.

#### GRaNPA: Calculating variable importance measures

We used the permutation approach to measure variable importance using the “ranger” package in R. This accuracy‐based approach uses the out‐of‐bag sample to calculate the importance of a specific variable. The importance is based on the difference in the prediction accuracy of out‐of‐bag sample and the prediction accuracy of out‐of‐bag sample while its variables have been randomly shuffled while all other variables kept the same.

#### GRN benchmarking against other networks/tools

Dorothea (Garcia‐Alonso *et al*, 2019; Holland *et al*, 2020a, 2020b): a resource containing TF‐target interactions. The connections are tagged by a confidence level based on the number of supporting evidence. The confidence levels range from A: highly reliable to B–D: curated and/or ChIP‐seq interactions to the lowest confidence level E: only computational support.

TRRUST (Han *et al*, 2018): a database of transcriptional regulatory networks for humans and mice. It has been constructed using text‐mining followed by manual curation. TRRUST v2 regulatory network for humans contains 795 TFs, 2,067 genes and 8,427 regulatory links. We did not use any weighing for the edges as it was not provided by TRRUST v2.

ChEA3 (Keenan *et al*, 2019): TF‐target gene libraries containing targets determined by ChIP‐seq experiments from ENCODE, ReMap and publications. It also contains co‐expression connections based on RNA‐seq data from resources like GREx and ARCHs4.

ANANSE (Xu *et al*, 2021): an enhancer‐based cell‐type‐specific network that can predict key transcription factors in cell fate determination. We used ANANSE macrophage‐specific network filtered by 0.8 probability for its links.

#### Assessing the cell‐type specificity of network models

To assess the cell‐type specificity of network models, we checked their performance on DE data from other cell‐types. In this analysis, we used naive macrophages, naive T‐cell and AML and evaluated their performance for DE data from GPR56‐positive versus GPR56‐negative AML, resting versus stimulated CD4‐positive follicular T‐cell (in 10 different subcell‐types) and naive‐macrophages versus 5‐h infected with Salmonella. We filtered genes using 0.1 adjusted *P*‐value and 1 absolute log fold change thresholds.

#### Enrichment of regulons among TF K/O data

We obtained cell‐type‐specific knockout (K/O) data for *NFKB1* in THP1‐derived macrophages (Somma *et al*, 2021), for *IRF8* in the human AML cell line MV4‐11 (Liss *et al*, 2021), and for *IRF1* and *IRF2* in primary human CD4^+^ T cells (Freimer *et al*, 2022). Differential expression analysis was performed using DESeq2 (Love *et al*, 2014), comparing all K/O conditions to their nontargeting controls. All genes significantly downregulated upon TF K/O (adjusted *P*‐value < 0.05 and fold‐change < 0), were considered as TF K/O affected genes. For each TF, we then quantified the enrichment of TF K/O affected genes among GRaNIE‐inferred target genes in the respective cell type using Fisher's exact test as implemented in the *GeneOverlap* package in R. Enrichments with at least five overlapping genes and Fisher's exact *P*‐value < 0.05 were considered significant.

#### Visualisation (Shiny App)

We provide a web application based on a Shiny App for interactive visualisation of the eGRNs for different cell types (https://apps.embl.de/grn/).

#### Gene set enrichment analysis

Preranked gene set enrichment analysis (GSEA, ranking based on log2 fold‐change) was performed using the Bioconductor/R package *fgsea* (preprint: Korotkevich *et al*, 2016). The M1 macrophage signature gene set was obtained from (Orecchioni *et al*, 2019).

#### GWAS enrichment

We tested whether the macrophage GRNs were enriched for genetic heritability of GWAS traits using stratified linkage disequilibrium score regression (S‐LDSC) (Finucane *et al*, 2015). 806 GWAS summary statistics that included participants of European descent were downloaded from the *GWAS Catalogue* (Buniello *et al*, 2019), harmonised and converted to the LDSC format as described on the LDSC Github repository (https://github.com/bulik/ldsc). We removed summary statistics with fewer than 10,000 individuals and fewer than 100,000 SNPs, because those studies were likely underpowered, leaving 442 traits. We created peak sets for each of the three macrophage GRNs by extracting the enhancer regions that were present in the GRNs after filtering for peak‐gene distance, TF‐peak false discovery rate (FDR) and gene differential expression effect size. We used 54 sets of general genomic features (downloaded from https://alkesgroup.broadinstitute.org/LDSCORE/, following Finucane *et al*, 2018) and a peak set based on all macrophage enhancers within 250 kb of genes as background regions. Adding these regions as a background ensures that the identified enrichments are not due to the general enrichment of heritability in (macrophage) enhancers near genes, but specifically in the enhancers that are part of the GRNs. We calculated the heritability enrichment P‐values and corrected them for multiple testing within each trait.

#### GRaNIE: GO enrichment of GRNs

The general enrichment analysis was run using *topGO* (v2.42.0) as part of the standard GRaNIE workflow, with the foreground being the genes in the filtered GRN, and the background being the genes within a predefined 250 kb neighbourhood of the peaks in the GRN. In more specific enrichment analyses such as those for the top transcription factors, which are ranked by their predictive capacity, the foreground is selected based on the genes a given TF is connected to within the network. Similarly, in community‐based enrichment analyses, the foreground is simply the genes that are classified to a given community. To calculate the enrichment, a Fisher test is used alongside the weight01 algorithm, which is a mixture of the “*elim*” and the “*weight*” algorithms introduced by (Alexa *et al*, 2006), to account for the GO hierarchy. Additionally, terms with less than 4 significantly annotated genes were omitted from the results in the figures. For the sake of better visualising the enriched terms, the figures are limited to the top 10 enriched terms per category. The full list of enriched terms can be found in Datasets EV7 and EV8.

#### Identifying targets for fine‐mapped GWAS‐SNPs using eGRNs

Fine‐mapped GWAS variants for autoimmune diseases were generated using probabilistic identification of causal SNPs (PICS) algorithm. These variants were downloaded from (i) previously published list and lifted to the *hg38* build (Farh *et al*, 2015) and (ii) data portal under the filename “PICS2‐GWAScat‐2020‐05‐22.txt.gz” from https://pics2.ucsf.edu for the hg38 build. We kept all variants with a PICS probability of greater than 50%. We identified their target genes by overlapping these variants with the peaks from different macrophage eGRNs and then using the peak‐gene links from the respective eGRNs to assign the target genes (Fig 5C). The full list can be found in Table 3.

## Author contributions

**Aryan Kamal:** Conceptualization; data curation; software; formal analysis; validation; investigation; visualization; methodology; writing – original draft; writing – review and editing. **Christian Arnold:** Conceptualization; data curation; software; formal analysis; validation; investigation; visualization; methodology; writing – original draft; writing – review and editing. **Annique Claringbould:** Conceptualization; data curation; formal analysis; validation; investigation; visualization; writing – original draft; writing – review and editing. **Nila H Servaas:** Data curation; formal analysis; validation; investigation; visualization. **Rim Moussa:** Data curation; software; formal analysis; methodology. **Maksim Kholmatov:** Data curation; formal analysis; validation; visualization. **Neha Daga:** Data curation; formal analysis; validation; investigation; visualization. **Daria Nogina:** Data curation; formal analysis. **Sophia Mueller‐Dott:** Conceptualization; data curation; software; formal analysis; investigation; methodology. **Armando Reyes‐Palomares:** Conceptualization; methodology. **Giovanni Palla:** Conceptualization. **Olga Sigalova:** Data curation; formal analysis. **Daria Bunina:** Conceptualization. **Caroline Pabst:** Resources. **Judith B Zaugg:** Conceptualization; software; supervision; funding acquisition; investigation; methodology; writing – original draft; project administration; writing – review and editing.

## Disclosure and competing interests statement

We declare that none of the authors have any competing interests. JBZ is an editorial advisory board member. This has no bearing on the editorial consideration of this article for publication.

## Supporting information



Appendix

Dataset EV1

Dataset EV2

Dataset EV3

Dataset EV4

Dataset EV5

Dataset EV6

Dataset EV7

Dataset EV8

Dataset EV9

Dataset EV10

## Data Availability

The methods implementation codes are available in the following GitLab repositories: (i) GRaNIE: https://git.embl.de/grp‐zaugg/GRaNIE; (ii) GRaNPA: https://git.embl.de/grp‐zaugg/GRaNPA.
