# Supplementary material for: GRaNIE and GRaNPA: inference and evaluation of enhancer‐mediated gene regulatory networks
Source: Mol Syst Biol. 2023 Apr 19;19(6):e11627. doi: 10.15252/msb.202311627 (PMC10258561; doi:10.15252/msb.202311627)
Supplement: Supplementary file 12 [file MSB-19-e11627-s011.pdf]

GRaNIE and GRaNPA: Inference and evaluation of enhancer-mediated gene regulatory networks

Aryan Kamal, Christian Arnold, Annique Claringbould, Nila Servaas, Rim Moussa, Maksim Kholmatov, Neha Daga, Daria Nogina, Sophia Mueller-Dott, Armando Reyes-Palomares, Giovanni Palla, Olga Sigalova, Daria Bunina, Caroline Pabst, and Judith Zaugg
DOI: 10.15252/msb.202311627

Corresponding author(s): Judith Zaugg (judith.zaugg@embl.de)

Review Timeline:

Submission Date:	27th Feb 23
Editorial Decision:	28th Feb 23
Revision Received:	23rd Mar 23
Editorial Decision:	29th Mar 23
Revision Received:	1st Apr 23
Accepted:	3rd Apr 23

Editor: Maria Polychronidou

Transaction Report:

(Note: With the exception of the correction of typographical or spelling errors that could be a source of ambiguity, letters and reports are not edited. Depending on transfer agreements, referee reports obtained elsewhere may or may not be included in this compilation. Referee reports are anonymous unless the Referee chooses to sign their reports.)

The reviewers' comments and authors' responses are not available with this article, as the initial review process took place with another journal.

1st Editorial Decision **28th Feb 2023**

Thank you once again for submitting your manuscript "GRaNIE and GRaNPA: Inference and evaluation of enhancer-mediated gene regulatory networks" to Molecular Systems Biology. We have now discussed the manuscript and the reviewers' comments from the other journal. We find that the topic fits well to the scope of MSB and that the presented methods seem relevant. Overall, we think that the remaining concerns of the reviewer from the submission at the other journal have been satisfactorily addressed. As such, I am glad to inform you that we would be happy to publish the study in Molecular Systems Biology, pending some editorial issues listed below.

Summary:

We have followed all the editorial guidelines (see below). In addition, we have made editorial changes to the text to improve clarity of the manuscript. For this, we have submitted a version of the manuscript in track-change mode.

- Please include 5 keywords.

1 - Gene regulatory networks
2 - Enhancers
3 - Transcriptional regulation
4 - Macrophage biology
5 - Multiomics data integration

- It would be better to list the "limitations of GRaNIE and GRaNPA" in a "Box", if you wish to keep the bullet point format (see an example of a Box here: https://www.embopress.org/doi/full/10.15252/msb.20178102). If you would prefer to keep this information in the discussion, the bullet point format should be removed.

Thank you for the suggestion, we agree that a box would be the best format. For now we have made box 1: limitations of GRaNIE and box 2: limitations of GRaNPA, but we are happy to put it into one box if that's better.

- We would recommend using the article type "Method".

Yes we agree.

- Please provide a .doc version of the manuscript text (including legends for the main figures) and individual production quality figure files for the main Figures (one file per figure).

We have submitted the manuscript as .doc one where we have cleaned all track-changes and a track-changed version where we recorded all the changes made after the acceptance (mostly editorial changes for clarifying the text).

- We have replaced Supplementary Information by the Expanded View (EV format). In this case, all additional figures can be included in a PDF called Appendix. Appendix figures should be labeled and called out as: "Appendix Figure S1, Appendix Figure S2... Appendix Table S1..." etc. Each legend should be below the corresponding Figure/Table in the Appendix. Please include a Table of Contents in the beginning of the Appendix. For detailed instructions regarding expanded view please refer to our Author Guidelines: http://msb.embopress.org/authorguide#expandedview.

We have done that.

- Tables S1-S10 should be provided Datasets EV1-EV10, labeled and called out as Dataset EV1, Dataset EV2 etc.). Please include in each .xls file, a separate tab with a brief description of the dataset.

We have done that.

- Please include a "Disclosure Statement and Competing Interests" in the main text.

We have done that.

- Please include a Data Availability section describing how the data, code etc. have been made available. The Data availability section needs to be formatted according to the example below:

The datasets and code produced in this study are available in the following databases:

- Chip-Seq data: Gene Expression Omnibus GSE46748 (https://www.ncbi.nlm.nih.gov/geo/query/acc.cgi?acc=GSE46748)

- Modeling computer scripts: GitHub (https://github.com/SysBioChalmers/GECKO/releases/tag/v1.0)

- [data type]: [full name of the resource] [accession number/identifier] ([doi or URL or identifiers.org/DATABASE:ACCESSION])

We have done that.

- Please provide a "standfirst text" summarizing the study in one or two sentences (approximately 250 characters), three to four "bullet points" highlighting the main findings and a "synopsis image" (550px width and max 400px height, jpeg format) to highlight the paper on our homepage.

GRaNIE builds enhancer-based gene regulatory networks using chromatin accessibility and RNA-seq data. GRaNPA assesses biological significance of GRNs and transcription factors. Together they provide insights into cell-type specific gene regulation.

1. GRaNIE builds gene regulatory networks that encompass transcription factors, regulatory regions, and genes, enabling a comprehensive view of gene regulation.
2. GRaNPA provides an unbiased evaluation method for cell-type specific GRNs by testing their ability to predict cell type specific differential expression.
3. GRaNPA can identify important transcription factors that drive differential expression, leading to insights into biological mechanisms.
4. GRaNIE and GRaNPA analysis identified PURA as a promising candidate for regulating pro-inflammatory macrophage polarization.

- All Materials and Methods need to be described in the main text. We would ask you to use 'Structured Methods', our new Materials and Methods format, which is mandatory for Methods (and Articles with a strong methodological focus). According to this format, the Material and Methods section should include a Reagents and Tools Table (listing key reagents, experimental models, software and relevant equipment and including their sources and relevant identifiers) followed by a Methods and Protocols section in which we encourage the authors to describe their methods using a step-by-step protocol format with bullet points, to facilitate the adoption of the methodologies across labs. More information on how to adhere to this format as well as downloadable templates (.doc or .xls) for the Reagents and Tools Table can be found in our author guidelines: https://www.embopress.org/page/journal/17444292/authorguide#textformate. An example of a Method paper with Structured Methods can be found here: https://www.embopress.org/doi/10.15252/msb.20178071.

We have done that.

- Please format the References according to the Molecular Systems Biology reference style i.e. ordered alphabetically and listing the first 10 authors followed by et al.

We have done that.

- For data quantification: please specify the name of the statistical test used to generate error bars and P values, the number (n) of independent experiments (specify technical or biological replicates) underlying each data point and the test used to calculate p-values in each figure legend. The figure legends should contain a basic description of n, P and the test applied. Graphs must include a description of the bars and the error bars (s.d., s.e.m.).

We have done that.

- Molecular Systems Biology supports formal data citations in the Reference list, to cite previously published datasets. In addition to citing the original papers that reported the data, we encourage you to also cite the relevant datasets directly in the Reference list. In the text, references to datasets are included as "Data ref: Smith et al, 2001" or "Data ref: NCBI Sequence Read Archive PRJNA342805, 2017". In the Reference list, data citations are very similar to normal literature references but must be labeled with "[DATASET]" at the end of the reference. For detailed instructions please refer to our Author Guidelines http://msb.embopress.org/authorguide#datacitation.

All the data we use is described in the original publications, so we have just cited the original studies.

- When you resubmit your manuscript, please download our CHECKLIST (https://bit.ly/EMBOPressAuthorChecklist) and include the completed form in your submission. *Please note* that the Author Checklist will be published alongside the paper

as part of the transparent process
(https://www.embopress.org/page/journal/17444292/authorguide#transparentprocess)

We have done that.

####################################

IMPORTANT: When you send your revision, we will require the following items:

1. the manuscript text in LaTeX, RTF or MS Word format

We have done that.

2. a letter with a detailed description of the changes made in response to the referees. Please specify clearly the exact places in the text (pages and paragraphs) where each change has been made in response to each specific comment given

We have done that.

3. three to four 'bullet points' highlighting the main findings of your study

We have done that.

4. a short 'blurb' text summarizing in two sentences the study (max. 250 characters)

We have done that.

5. a 'thumbnail image' (550px width and max 400px height, Illustrator, PowerPoint or jpeg format), which can be used as 'visual title' for the synopsis section of your paper.

We have done that.

6. Please include an author contributions statement after the Acknowledgements section (see https://www.embopress.org/page/journal/17444292/authorguide#manuscriptpreparation)

We have done that.

7. Please complete the CHECKLIST available at (https://bit.ly/EMBOPressAuthorChecklist).

Please note that the Author Checklist will be published alongside the paper as part of the transparent process (https://www.embopress.org/page/journal/17444292/authorguide#transparentprocess).

We have done that.

8. When assembling figures, please refer to our figure preparation guideline in order to ensure proper formatting and readability in print as well as on screen:

https://bit.ly/EMBOPressFigurePreparationGuideline

See also figure legend guidelines:
https://www.embopress.org/page/journal/17444292/authorguide#figureformat

We have modified the figure legends to fit this formatting

9. Please note that corresponding authors are required to supply an ORCID ID for their name upon submission of a revised manuscript (EMBO Press signed a joint statement to encourage ORCID adoption).
(https://www.embopress.org/page/journal/17444292/authorguide#editorialprocess)

Currently, our records indicate that the ORCID for your account is 0000-0001-8324-4040.

That's correct

1st Revision - Editorial Decision 29th Mar 2023

Thank you for sending us your revised manuscript. Before we formally accept the study for publication we would ask you to address a few remaining editorial issues:

All editorial and formatting issues were resolved by the authors.

2nd Revision - Editorial Decision **3rd Apr 2023**

Thank you again for sending us your revised manuscript. We are now satisfied with the modifications made and I am pleased to inform you that your paper has been accepted for publication.

EMBO Press Author Checklist

Corresponding Author Name: Judith Zaugg	
Journal Submitted to: Molecular Systems Biology	
Manuscript Number: MSB-2023-11627	

USEFUL LINKS FOR COMPLETING THIS FORM
The EMBO Journal - Author Guidelines
EMBO Reports - Author Guidelines
Molecular Systems Biology - Author Guidelines
EMBO Molecular Medicine - Author Guidelines

Reporting Checklist for Life Science Articles (updated January
This checklist is adapted from Materials Design Analysis Reporting (MDAR) Checklist for Authors. MDAR establishes a minimum set of requirements in transparent reporting in the life sciences (see Statement of Task: 10.31222/osf.io/9sm4x). Please follow the journal's guidelines in preparing your
Please note that a copy of this checklist will be published alongside your article.

Abridged guidelines for figures
1. Data
The data shown in figures should satisfy the following conditions:
- the data were obtained and processed according to the field’s best practice and are presented to reflect the results of the experiments in an accurate and unbiased manner.
- ideally, figure panels should include only measurements that are directly comparable to each other and obtained with the same assay.
- plots include clearly labeled error bars for independent experiments and sample sizes. Unless justified, error bars should not be shown for technical
- if n<5, the individual data points from each experiment should be plotted. Any statistical test employed should be justified.
- Source Data should be included to report the data underlying figures according to the guidelines set out in the authorship guidelines on Data

2. Captions
Each figure caption should contain the following information, for each panel where they are relevant:
- a specification of the experimental system investigated (eg cell line, species name).
- the assay(s) and method(s) used to carry out the reported observations and measurements.
- an explicit mention of the biological and chemical entity(ies) that are being measured.
- an explicit mention of the biological and chemical entity(ies) that are altered/varied/perturbed in a controlled manner.
- the exact sample size (n) for each experimental group/condition, given as a number, not a range;
- a description of the sample collection allowing the reader to understand whether the samples represent technical or biological replicates (including how many animals, litters, cultures, etc.).
- a statement of how many times the experiment shown was independently replicated in the laboratory.
- definitions of statistical methods and measures:
  - common tests, such as t-test (please specify whether paired vs. unpaired), simple χ2 tests, Wilcoxon and Mann-Whitney tests, can be unambiguously identified by name only, but more complex techniques should be described in the methods section;
  - are tests one-sided or two-sided?
  - are there adjustments for multiple comparisons?
  - exact statistical test results, e.g., P values = x but not P values < x;
  - definition of ‘center values’ as median or average;
  - definition of error bars as s.d. or s.e.m.

Please complete ALL of the questions below.
Select "Not Applicable" only when the requested information is not relevant for your study.

Materials

Newly Created Materials	Information included in the manuscript?	In which section is the information available? (Reagents and Tools Table, Materials and Methods, Figures, Data Availability Section)
New materials and reagents need to be available; do any restrictions apply?	Not Applicable	

Antibodies	Information included in the manuscript?	In which section is the information available? (Reagents and Tools Table, Materials and Methods, Figures, Data Availability Section)
For **antibodies** provide the following information:<br>- Commercial antibodies: RRID (if possible) or supplier name, catalogue number and or/clone number<br>- Non-commercial: RRID or citation	Not Applicable	

DNA and RNA sequences	Information included in the manuscript?	In which section is the information available? (Reagents and Tools Table, Materials and Methods, Figures, Data Availability Section)
**Short novel DNA or RNA including primers, probes**: provide the sequences.	Not Applicable	

Cell materials	Information included in the manuscript?	In which section is the information available? (Reagents and Tools Table, Materials and Methods, Figures, Data Availability Section)
**Cell lines:** Provide species information, strain. Provide accession number in repository **OR** supplier name, catalog number, clone number, and/**OR** RRID.	Not Applicable	
**Primary cultures:** Provide species, strain, sex of origin, genetic modification status.	Not Applicable	
Report if the cell lines were recently **authenticated** (e.g., by STR profiling) and tested for mycoplasma contamination.	Not Applicable	

Experimental animals	Information included in the manuscript?	In which section is the information available? (Reagents and Tools Table, Materials and Methods, Figures, Data Availability Section)
**Laboratory animals or Model organisms:** Provide species, strain, sex, age, genetic modification status. Provide accession number in repository **OR** supplier name, catalog number, clone number, **OR** RRID.	Not Applicable	
**Animal observed in or captured from the field:** Provide species, sex, and age where possible.	Not Applicable	
Please detail **housing and husbandry conditions**.	Not Applicable	

Plants and microbes	Information included in the manuscript?	In which section is the information available? (Reagents and Tools Table, Materials and Methods, Figures, Data Availability Section)
**Plants:** provide species and strain, ecotype and cultivar where relevant, unique accession number if available, and source (including location for collected wild specimens).	Not Applicable	
**Microbes:** provide species and strain, unique accession number if available, and source.	Not Applicable	

Human research participants	Information included in the manuscript?	In which section is the information available? (Reagents and Tools Table, Materials and Methods, Figures, Data Availability Section)
If collected and within the bounds of privacy constraints report on age, sex and gender or ethnicity for all study participants.	Not Applicable	

Core facilities	Information included in the manuscript?	In which section is the information available? (Reagents and Tools Table, Materials and Methods, Figures, Data Availability Section)
If your work benefited from core facilities, was their service mentioned in the acknowledgments section?	Not Applicable	

Design

Study protocol	Information included in the manuscript?	In which section is the information available? (Reagents and Tools Table, Materials and Methods, Figures, Data Availability Section)
If study protocol has been **pre-registered, provide DOI in the manuscript**. For clinical trials, provide the trial registration number **OR** cite DOI.	Not Applicable	
Report the **clinical trial registration number** (at ClinicalTrials.gov or equivalent), where applicable.	Not Applicable	

Laboratory protocol	Information included in the manuscript?	In which section is the information available? (Reagents and Tools Table, Materials and Methods, Figures, Data Availability Section)
Provide DOI OR other citation details if **external detailed step-by-step protocols** are available.	Not Applicable	

Experimental study design and statistics	Information included in the manuscript?	In which section is the information available? (Reagents and Tools Table, Materials and Methods, Figures, Data Availability Section)
Include a statement about **sample size** estimate even if no statistical methods were used.	Not Applicable	
Were any steps taken to minimize the effects of subjective bias when allocating animals/samples to treatment (e.g. **randomization procedure**)? If yes, have they been described?	Not Applicable	
Include a statement about **blinding** even if no blinding was done.	Not Applicable	
Describe **inclusion/exclusion criteria** if samples or animals were excluded from the analysis. Were the criteria pre-established?<br><br>If sample or data points were omitted from analysis, report if this was due to attrition or intentional exclusion and provide justification.	Not Applicable	
For every figure, are **statistical tests** justified as appropriate? Do the data meet the assumptions of the tests (e.g., normal distribution)? Describe any methods used to assess it. Is there an estimate of variation within each group of data? Is the variance similar between the groups that are being statistically compared?	Yes	Materials and Methods

Sample definition and in-laboratory replication	Information included in the manuscript?	In which section is the information available? (Reagents and Tools Table, Materials and Methods, Figures, Data Availability Section)
In the figure legends: state number of times the experiment was **replicated** in laboratory.	Not Applicable	
In the figure legends: define whether data describe **technical or biological replicates**.	Not Applicable	

Ethics

Ethics	Information included in the manuscript?	In which section is the information available? (Reagents and Tools Table, Materials and Methods, Figures, Data Availability Section)
Studies involving **human participants**: State details of **authority granting ethics approval** (IRB or equivalent committee(s), provide reference number for approval.	Not Applicable	
Studies involving **human participants**: Include a statement confirming that **informed consent** was obtained from all subjects and that the experiments conformed to the principles set out in the WMA Declaration of Helsinki and the Department of Health and Human Services Belmont Report.	Not Applicable	
Studies involving **human participants:** For publication of **patient photos**, include a statement confirming that consent to publish was obtained.	Not Applicable	
Studies involving experimental **animals**: State details of **authority granting ethics approval** (IRB or equivalent committee(s), provide reference number for approval. Include a statement of compliance with ethical regulations.	Not Applicable	
Studies involving **specimen and field samples:** State if relevant **permits** obtained, provide details of authority approving study; if none were required, explain why.	Not Applicable	

Dual Use Research of Concern (DURC)	Information included in the manuscript?	In which section is the information available? (Reagents and Tools Table, Materials and Methods, Figures, Data Availability Section)
Could your study fall under dual use research restrictions? Please check biosecurity documents and list of **select agents and toxins** (CDC): https://www.selectagents.gov/sat/list.htm	Not Applicable	
If you used a select agent, is the security level of the lab appropriate and reported in the manuscript?	Not Applicable	
If a study is subject to dual use research of concern regulations, is the name of the **authority granting approval and reference number** for the regulatory approval provided in the manuscript?	Not Applicable	

Reporting

The MDAR framework recommends adoption of discipline-specific guidelines, established and endorsed through community initiatives. Journals have their own policy about requiring specific guidelines and recommendations to complement MDAR.

Adherence to community standards	Information included in the manuscript?	In which section is the information available? (Reagents and Tools Table, Materials and Methods, Figures, Data Availability Section)
State if relevant guidelines or checklists (e.g., **ICMJE, MIBBI, ARRIVE, PRISMA**) have been followed or provided.	Not Applicable	
For **tumor marker prognostic studies,** we recommend that you follow the **REMARK** reporting guidelines (see link list at top right). See author guidelines, under 'Reporting Guidelines'. Please confirm you have followed these guidelines.	Not Applicable	
For **phase II and III randomized controlled trials**, please refer to the **CONSORT** flow diagram (see link list at top right) and submit the CONSORT checklist (see link list at top right) with your submission. See author guidelines, under 'Reporting Guidelines'. Please confirm you have submitted this list.	Not Applicable	

Data Availability

Data availability	Information included in the manuscript?	In which section is the information available? (Reagents and Tools Table, Materials and Methods, Figures, Data Availability Section)
Have **primary datasets** been deposited according to the journal's guidelines (see 'Data Deposition' section) and the respective accession numbers provided in the Data Availability Section?	Not Applicable	
Were **human clinical and genomic datasets** deposited in a public access-controlled repository in accordance to ethical obligations to the patients and to the applicable consent agreement?	Not Applicable	
Are **computational models** that are central and integral to a study available without restrictions in a machine-readable form? Were the relevant accession numbers or links provided?	Yes	Data Availability Section
If publicly available data were reused, provide the respective **data citations in the reference list**.	Yes	Materials and Methods
